# Disentangling local, metapopulation, and cross-community sources of stabilization and asynchrony in metacommunities

**DOI:** 10.1002/ecs2.3078

**Published:** 2020-04-21

**Authors:** Matthew Hammond, Michel Loreau, Claire de Mazancourt, Jurek Kolasa

**Affiliations:** 1 Department of Biology, McMaster University, 1280 Main Street West, Hamilton Ontario L8S 4K1 Canada; 2 Centre for Biodiversity Theory and Modelling, Theoretical and Experimental Ecology Station, CNRS, 2 Route du CNRS, 09200 Moulis, France

**Keywords:** asynchrony, community, diversity, diversity-stability, metacommunity, metapopulation, partitioning, stability, variability

## Abstract

Asynchronous fluctuations of populations are essential for maintaining stable levels of bio-mass and ecosystem function in landscapes. Yet, understanding the stabilization of metacommunities by asynchrony is complicated by the existence of multiple forms of asynchrony that are typically studied independently: Community ecologists, for instance, focus on asynchrony within and among local communities, while population ecologists emphasize asynchrony of populations in metapopulations. Still, other forms of asynchrony, such as that which underlies the spatial insurance effect, are not captured by any existing analytical frameworks. We therefore developed a framework that would in one analysis unmask the stabilizing roles of local communities and metapopulations and so unify these perspectives. Our framework shows that metacommunity stabilization arises from one local and two regional forms of asynchrony: (1) asynchrony among species of a local community, (2) asynchrony among populations of a metapopulation, and (3) cross-community asynchrony, which is between different species in different local communities and underlies spatial insurance. For each type of stabilization, we derived links to diversity indices and associated diversity-stability relationships. We deployed this framework in a set of rock pool invertebrate metacommunities in Discovery Bay, Jamaica, to partition sources of stabilization and test their dependence on diversity. Cross-community asynchrony was the dominant form of stabilization, accounting for >60% of total metacommunity stabilization despite being undetectable with existing frameworks. Environmental variation influenced types of stabilization through different mechanisms. pH and dissolved oxygen, for example, increased asynchrony by decorrelating local species, while salinity did so by changing the abundance structure of metapopulations. Lastly, all types of asynchrony depended strongly on different types of diversity (alpha, metapopulation, and beta diversity drove local, metapopulation, and cross-community asynchrony, respectively) to produce multiple diversity-stability relationships within metacommunities. Our new partition of metacommunity dynamics highlights how different elements—from local communities to metapopulations—combine to stabilize metacommunities and depend critically on contrasting environmental regimes and diversities. Understanding and balancing these sources of stability in dynamic landscapes is a looming challenge for the future. We suggest that synthetic frameworks which merge ecological perspectives will be essential for grasping and safeguarding the stability of natural systems.

## Introduction

Community-level biomass or abundance varies over time and governs the rise and fall of ecosystem functions in landscapes. Such system-level fluctuations are stabilized when components (e.g., species) fluctuate asynchronously so that declines in one component are compensated by increases in another (Doak et al. 1998, Yachi and Loreau 1999, Schindler et al. 2015). Because asynchrony reduces variation of community or ecosystem properties, it is important for ensuring their reliability. Alaskan salmon returns, for example, are stabilized by the existence of hundreds of uncoupled populations (Schindler et al. 2010). Tallgrass prairie biomass is similarly stabilized where fire and grazing create a mosaic of asynchronous patches (McGranahan et al. 2016). In turn, biomass stabilization can be crucial for stabilizing ecosystem functions like net primary production (Wilcox et al. 2017).

Recent work has isolated the mechanisms by which asynchrony stabilizes natural systems. Support for the insurance hypothesis (Yachi and Loreau 1999) highlights the stabilizing effect of asynchronous species responses to environmental fluctuations (Leary and Petchey 2009, Hector et al. 2010, Loreau 2010). Confirmed portfolio effects (sensu Doak et al. 1998, Tilman 1999), meanwhile, demonstrate the power of diversity to stabilize communities or functional groups when species dynamics are weakly correlated (Bai et al. 2004, Cardinale et al. 2012). But while stabilization by asynchrony is well understood in local communities (Thibaut and Connolly 2013), there is an urgent conservation need to scale that understanding up to metacommunities (Wang and Loreau 2014).

In a recent advance, Wang and Loreau (2014) partitioned the variability of total metacommunity biomass or abundance—gamma variability (γ_CV_) —into local and regional components representing the variability of local communities (α_CV_) and asynchrony among those communities (β). Their approach has rapidly become the most common in metacommunity asynchrony research and has underscored the importance of spatial heterogeneity in stabilizing metacommunity biomass and ecosystem function (McGranahan et al. 2016, Wilcox et al. 2017). But despite this progress, two barriers—one analytical and the other conceptual— prevent a deeper understanding of stabilization at the metacommunity scale.

The analytical barrier is that the main local community framework used to date (Wang and Loreau 2014) does not capture some forms of regional asynchrony that interest ecologists. Asyn-chrony among populations of a metapopulation, for example, helps to stabilize overall metacom-munity biomass (Wilcox et al. 2017) and is critical for species persistence in landscapes (Anderson et al. 2015, Schindler et al. 2015). But this form of asynchrony is only implicit in the local community framework (Wilcox et al. 2017), leaving its contribution to stability at the metacommunity scale unquantified. Another form of asynchrony overlooked by current frameworks is that which underlies the spatial insurance hypothesis (Yachi and Loreau 1999), wherein different species occupying different patches fluctuate asynchronously and disperse to maintain ecosystem function (Gonzalez et al. 2009).

**Fig. 1:**
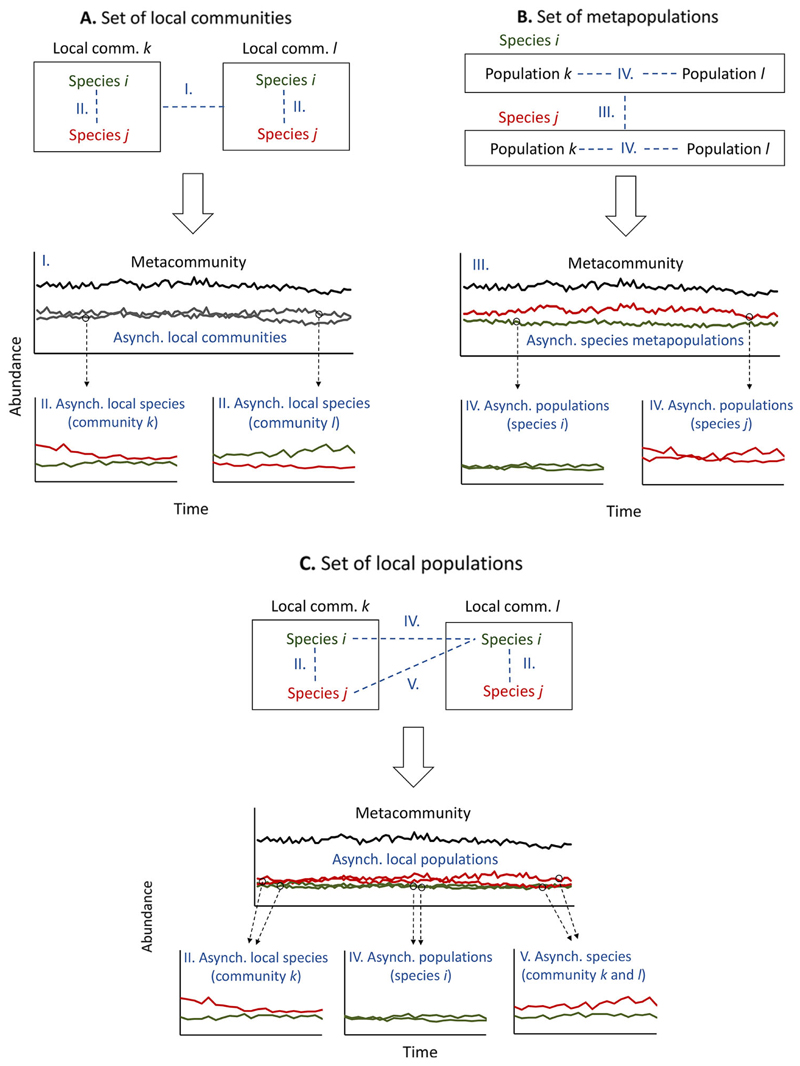
Three views of a metacommunity and their associated forms of asynchrony. Viewing metacommunities as (A) a set of local communities emphasizes asynchrony among local communities (type I asynchrony, blue dashed lines) and among species within local communities (type II), both of which stabilize total metacommunity abundance. (B) But viewed as a set of metapopulations, focus is on asynchrony among metapopulations (type III) and among populations of a metapopulation (type IV). (C) Here, we view the metacommunity as a set of local populations (species i in local community *k*). This bridges the local community and metapopulation perspectives to partition stabilizing asynchrony from species within local communities (type II), from populations within metapopulations (type IV), and from a cross-community form of asynchrony that occurs between different species inhabiting different local communities (type V).

The above gaps may be seen to result from a conceptual problem: The form of asynchrony measured depends on the organizational hierarchy used to conceptualize and study a metacom-munity (Fig. 1). Viewed as a set of local communities (Fig. 1A), for instance, the meta-community is stabilized by asynchrony among local communities (which we call type I asyn-chrony) and asynchrony of species within those local communities (type II; Wang and Loreau 2014). But if viewed (equally validly) as a set of metapopulations (Fig. 1B), it is stabilized by asynchrony among species metapopulations (type III) and asynchrony of populations within those metapopulations (type IV).

Progress in stability research depends on bringing these overlapping metacommunity perspectives together in a single frame of reference. Wang et al. (2019) made an important step in this direction by relating the local community and metapopulation hierarchies in an analytical framework. However, the approach does not reconcile local communities and metapopulations in a single analysis to give their independent contributions to metacommunity stability. Nor does it capture the fifth form of asynchrony (type V)— among different species in different local communities—that is the generative mechanism for spatial insurance (Loreau et al. 2003, Gonzalez et al. 2009).

Here, we present a new perspective on meta-community stabilization that overcomes the analytical and conceptual barriers left unad-dressed by past approaches (Fig. 1C). Viewing the metacommunity as a set of asynchronous local populations (i.e., population of species *i* in local community *k*) allows a highly resolved view of metacommunity dynamics (e.g., Gouhier et al. 2010). Moreover, it lets us partition asynchronies that would be hidden if the meta-community was analyzed as a set of local communities or a set of metapopulations. On the conceptual front, the approach unifies the local community and metapopulation hierarchies in a single analytical partition by including elements of each.

The resulting framework exposes how metacommunities are stabilized by one local-scale and two regional-scale forms of asynchrony—among local species (type II), among populations of a metapopulation (type IV), and among different species in different communities (type V), which we call cross-community asynchrony. Notably, these forms are wholly consistent with the definition of metacommunity dynamics (cf. Holyoak et al. 2005:9) as including a local community component (e.g., type II), a spatial component (e.g., type IV), and a community x spatial component (e.g., type V). A further advantage is that because the framework works at the resolution of species populations, it offers ties to biological mechanism and diversity that other frameworks do not (see Box 1 for details).

The framework thus has strong potential for synthesizing community and population ecology as well as exposing the stabilizing roles of diversity. Moreover, its application to empirical data— rock pool metacommunities here—should help to resolve the complex stabilization of ecosystems that emerges over many lower levels of organization (Proulx et al. 2010). It therefore offers a tantalizing step toward a full accounting of temporal stability at the metacommunity scale.

### Analytical framework: Disentangling stabilization by local communities, metapopulations, and more

Stabilization here is the reduction of variability at the metacommunity scale due to asynchrony. Analyzing the metacommunity as a set of local populations is the key to partitioning stabilizing asynchrony from both the local and metapopulation hierarchies. We define a local population *ik* as the individuals of species *i* living in a sampled local community *k*, though we recognize that these may not constitute a population in the demographic sense. As shown in Fig. 1C, focusing on local populations is the only approach that avoids an intermediate hierarchical level (e.g., local communities which are aggregates of local species) to expose all intra-and interspecific stabilization occurring at the population level.

Using local populations as the unit of analysis, we can quantify total stabilization from local population asynchrony, x. This is the degree to which variability of metacommunity biomass (gamma) is reduced by asynchrony among all local populations in the metacommunity (i.e., between all populations living in all local communities or, equivalently, between all populations of all species; see Table 1 for formulae and Appendix S1 for derivations).

Total stabilization reduces variability of meta-community biomass or abundance as

(1) $$\gamma_{\text{CV}}=\iota_{\text{CV}}-\omega ,$$

where *γ*_CV_ is the squared coefficient of variation (CV^2^) of metacommunity biomass or abundance (Wang and Loreau 2014). ι_CV_ is a weighted and squared average variability of all populations in the meta-community. It is also the value of *γ*_CV_ when all local populations are perfectly synchronized (Appendix S1: Eq. S6).

There are just three forms of asynchrony that can occur among local populations to stabilize the metacommunity (Fig. 1C). Total stabilization (x) thus splits into three components corresponding to the different pairings of populations and covariances possible (Appendix S1: Fig. S1):

(2) $$\omega \;=\;\delta \;+\;\beta_{\text{mp}}+\beta_{\text{CC}},$$

δ measures local stabilization or stabilization due to type I asynchrony among local species (species *i* with *j* in local community *k*). It is equivalent to within-community stabilization in the local community hierarchy (Fig. 1A) and Wang and Lor-eau’s (2014) additive partition (see Appendix S2). β_mp_ measures metapopulation stabilization or stabilization from type II asynchrony among populations in metapopulations (species *i* in local communities *k* and *l*). It is equivalent to within-species stabilization in the metapopulation hierarchy (Fig. 1B) and an additive version of Wang et al.’s (2019) partition (see Appendix S2). Lastly, *β*_CC_ quantifies cross-community stabilization from type V asynchrony between different species in different local communities (species *i* in local community *k* with species *j* in local community *l*). This source of stability reflects the degree to which contrasting dynamics of species spread across the landscape reduces metacommunity variability and is notably masked in existing hierarchical frameworks (Appendix S2: Fig. S1). Because the same asynchrony mechanism underlies the spatial insurance hypothesis, β_cc_ sheds new light on how species diversity and environmental heterogeneity interact to stabilize metacommunities.

Metacommunity biomass or abundance is stabilized whenever there is asynchrony among local populations in the metacommunity. We can express this asynchrony as 1 – φ_pop_, where φ is Loreau and de Mazancourts (2008) dimensionless measure of synchrony. Doing so, we find that stabilization (ω) depends on the average variability of local populations (ι_CV_) and their asynchrony in the metacommunity (Appendix S3):

(3) $$\omega \;=\;\left(1-\varphi_{\text{pop}}\right)\iota_{\text{CV}}.$$

Ecologists often study asynchrony as opposed to the resulting reduction of metacommunity variability (Thibaut and Connolly 2013, Hautier et al. 2014). For these applications, we can apply the same additive partition of stabilization (Eq. 2) to partition asynchrony (Appendix S3). We find population asynchrony (1 – φ_pop_) to be a composite of asynchrony from local (δ/ι_CV_), metapopulation (β_mp_/ι_CV_), and cross-community pairs of populations (β_cc_/ι_CV_):

(4) $$1-\varphi_{\text{pop}}=\frac{\delta }{\iota_{\text{CV}}}+\frac{\beta_{\text{mp}}}{\iota_{\text{CV}}}+\frac{\beta_{\text{cc}}}{\iota_{\text{CV}}}.$$

These components change with the degree of correlation between populations and enable deeper analysis of asynchrony in metacommunities.

### Stabilization of rock pool metacommunities

We illustrate our analytical framework in a set of tropical rock pool metacommunities. This system has been well-studied and has many positive attributes for testing metacommunity theory, such as high species diversity, discrete, identifiable local communities, and relatively independent annual samples of community composition and structure (Kolasa and Romanuk 2005). It thus offers a clear and well-resolved system for testing our framework. Our main goal is to understand how forms of stabilizing asynchrony that were previously overlooked or considered separately combine to stabilize metacommunities. In specific terms, stabilized metacommunity biomass or abundance has implications for sustaining generalist predators in rock pools (e.g., crab larvae Sesarma miersii Rathburn 1897) and smoothing ecosystem processes (e.g., primary productivity; Wilcox et al. 2017). But as a broader exploration of stabilization pathways, we ask:

1. Is metacommunity abundance most stabilized by local communities, metapopulations, or cross-community combinations of species?
2. What environmental factors drive the various forms of stabilization?
3. How do different forms of diversity influence stabilization at the metacommunity scale?

Through a unifying approach, our findings highlight multiple paths by which diversity stabilizes metacommunities from local to regional scales.

## Methods

### Study system and sampling

We sampled 49 coastal rock pools near Discovery Bay Marine Laboratory, University of the West Indies, on the northern coast of Jamaica (18°28′ N, 77°25′ W) over fourteen annual surveys (1989–2003). Pools lie on a 25 m radius section of fossil reef within 1 m of the nearest neighbor, on average, and no further than 10 m from the ocean. Pools have volumes ranging from 0.5 to 78.4 L and are refilled by precipitation, ocean spray, and, for a few, occasionally large ocean tides. Seventy-eight invertebrate species occur in the system and disperse as propagules transported by wind, ocean spray, animal vectors, and overflow after heavy rainfall (Sciullo and Kolasa 2012). We confined analyses to the 26 most abundant species. These species are those with densities of more than five individuals per pool and constitute 99% of all individuals. They therefore represent the diversity that contributes most to total metacommunity abundance and its stabilization by asynchrony. Analyzed species included ostracods (8 species), copepods (6), cladocerans (3), worms (5), aquatic insects (3), and other crustaceans (1).

We sampled invertebrate communities at low tide in December or January of a sampling year, with the exception of an additional June 1997 sampling. We withdrew 0.5 L of water after stirring the pool to dislodge organisms from rock walls and homogenize contents. Each sample was filtered through 63-μm mesh to isolate invertebrates, which were immediately preserved in 50% ethanol. Community samples were sorted, identified, and counted by microscope. Rock pools could not be sampled when pool drying caused volumes to fall below 0.5 L and were recorded as blanks (see below for data treatment). Environmental variables including temperature, salinity, dissolved oxygen, pH, and chlorophyll-a concentration (a proxy for biological productivity) were measured with multiprobe sondes (DataSonde, Yellow Springs Instruments, or Hydrolab). Data were available for 8–11 of the survey years, except for chlorophyll a which was measured on six annual surveys.

### Identification of replicate metacommunities

We identified seven subsystems within the rock pool landscape to serve as replicate metacommunities. Metacommunities could not be identified based on rates of organism dispersal as these are mostly unknown—a common deficiency in ecological data sets. Past work in the same system, however, indicates that dispersal is common (Sciullo and Kolasa 2012) and that its effects on local communities decay with distance (Pandit et al. 2009). We therefore delineated metacommunities based on their spatial clustering in the landscape. This approach assumes only that closer pools exchange more dispersers —and so form a more integrated system—than those that are farther apart and hence less connected by dispersal. But because identified subsystems may nonetheless still exchange organisms, it is important to recognize that they may not be completely independent. Still, the chosen systems provide a reasonable snapshot of clusters of sites that are more likely to exchange organisms on account of their proximity.

We used cluster analysis with complete linkage to group pools by geographic position in the *X*, *Y*, and *Z* (height above sea level) dimensions. The number of statistically justified clusters was determined by the elbow in the amalgamation schedule. Seven clusters were advanced as putative metacommunities. Metacommunities ranged in number of local communities (pools) from 3 to 25 (mean – 7.0 ±8.1 standard deviation [SD]) and in regional richness from 15 to 26 species (mean — 20.7 ± 4.3). Metacommunities spanned a range of environmental influences, from low-lying seaward pools to high-lying pools close to the leading edge of landward vegetation.

### Statistical analysis

Stabilization and asynchrony were analyzed at interannual timescales—the sampling frequency of data. Our analyses therefore omit any sub-annual asynchrony exhibited by pool organisms. We note, however, that even though stabilization can occur over multiple timescales (Downing et al. 2008), interannual fluctuations are a large source of variation in coastal ecosystems and can be an important timescale for stabilization.

Dried up rock pools that could not be sampled and were recorded as blank data entries constituted <10% of total observations. Since these blanks introduce errors when partitioning variability, we replaced them with zeros which assumes that no living, adult invertebrates occur in a dry pool. Stabilization and asynchrony metrics in Table 1 were calculated from rock pool density data for each replicate metacommunity. We used Statistica 8.0 software (StatSoft 2007) to test for differences in δ, β_mp_, and β_cc_ asynchrony with one-way ANOVAs and Tukey’s post hoc tests. Data were log- or square-root-transformed when necessary to meet parametric assumptions. Nonparametric Kruskal-Wallis tests were used if parametric assumptions could not be met. Because environmental variables were measured with differing frequencies, we calculated temporal means or CVs of a variable for each pool. These measures therefore summarized the long-term characteristics of pools (e.g., high salinity) as opposed to their instantaneous conditions. We employed standard and forward-step bivariate regressions to test for environmental drivers of stabilization.

We also used the CV-based formulae in Table 1 to calculate benchmark values of *γ*_CV_, δ, β_mp_, and β_cc_ for theoretical metacommunities with different statistical properties. We assessed, for instance, *γ*_CV_ values for (1) zero correlation between populations (ρ_*ik.jl*_ = 0), (2) even local populations (*p*_*ik*_ = 1/N_pop_, where N_pop_ is the number of populations in the metacommunity), and (3) even populations with zero correlation.

We used one-way ANOVA to test whether types of stabilization differed in terms of relative abundances and variability of population pairs (*p*_*ik*_ *p*_*jl*_ and CV_*ik*_CV_*jl*_, respectively). We used an alternative method to compare mean pairwise correlation (ρ_*ik.jl*_) between stabilization types because means of correlation coefficients are biased by the number of elements in the correlation matrix (Loreau and de Mazancourt 2008). Corrections for this bias exist (Loreau and de Mazancourt 2008, Gross et al. 2014, Blüthgen et al. 2016) but apply to an entire correlation matrix and not the local, metapopulation, and cross-community subsets we averaged. We therefore used random subsampling to keep the number of correlations constant across groups (types of stabilization or asynchrony). For each meta-community, we retained all *N* correlation coefficients for the smallest group and compared these to *N* correlations randomly selected from the larger groups.

We contrasted regressions of diversity and asynchrony in rock pool metacommunities with null cases of low and high correlation among metacommunity populations. These cases represent the extremes of uncorrelated and correlated responses to environmental fluctuations, respectively. We simulated uncorrelated responses by randomizing the order of time series values for each population. Correlated responses were simulated by aligning time series values in rank order within local communities, within metapop-ulations, or within the whole metacommunity to be as correlated as possible given time series values. We then explored a wider range of interpop-ulation correlation levels by using equations in Table 1 to calculate stabilization values for different correlation values from ρ_*ik.jl*_ = 0 to 1.

## Results

### Stabilization of rock pool metacommunities

A varying environment drove the temporal variability of populations in rock pool metacommunities (ι_CV_). Temperature variability (CV) was the primary environmental factor retained by stepwise multiple regression (*R* ^2^ = 0.70, *F*_1,5_ = 11.57, *P* = 0.019). But stabilization from local, metapopulation, and cross-community populations reduced variability at the metacommunity scale substantially by 74.9% ± 10.3 (SD). Comparisons with theoretical benchmarks showed stabilization to be close to the 97.9% ±1.2 reduction expected if all populations were even and uncorrelated (Appendix S6: Fig. S1). Moreover, our measures revealed 1.6 times more stabilization than would be detected within the local community hierarchy (i.e., using Wang and Loreau’s [2014] framework). This was because δ, β_mp_, and β_cc_ captured the stabilizing effects of all asynchronous populations in contrast to hierarchical frameworks that incompletely capture these effects (Appendix S2: Fig. S1).

**Fig. 2:**
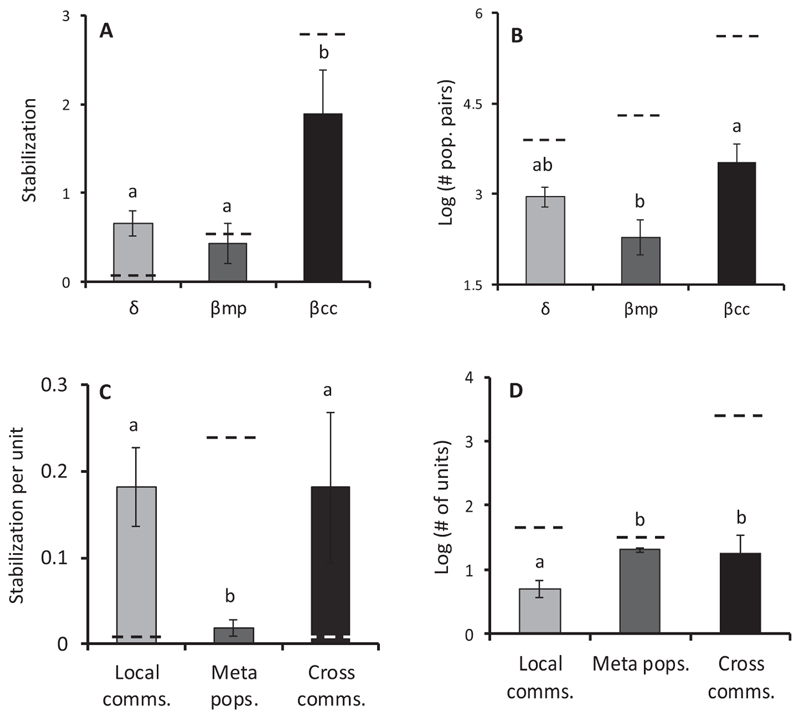
(A) Mean stabilization of gamma variability arising from local communities (δ), metapopulations (β_mp_), and cross-communities (β_cc_) in rock pool metacommunities. (B) Mean number of population pairs contributing asynchrony to δ, β_mp_, and β_cc_. (C) Stabilization per sampling unit in the metacommunity, that is per local community for δ, per metapopulation for β_mp_, and per local community pair for β_cc_. (D) Mean number of local communities, metapopulations, and local community pairs (cross-communities) represented in metacommunities. Dashed line indicates value for whole landscape of rock pools. Significant differences (P < 0.05) of raw or log-transformed values indicated by a and b groupings.

**Fig. 3:**
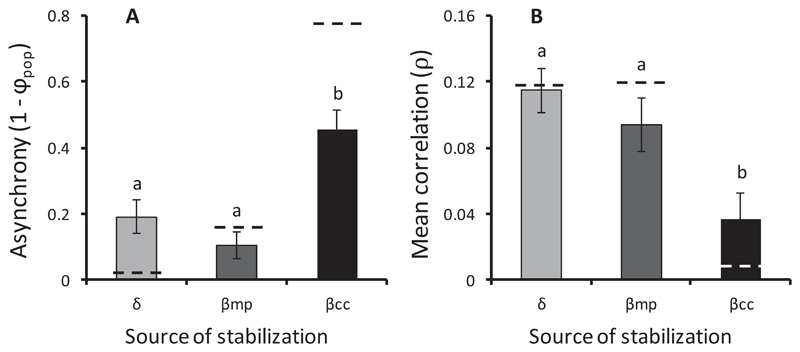
Asynchrony and correlation underlying stabilization by δ, β_mp_, and β_cc_. (A) Local, metapopulation, and cross-community components of asynchrony, calculated according to Eq. 4. (B) Unbiased mean correlation of local, metapopulation, and cross-community population pairs. Dashed line indicates value for whole landscape of rock pools.

Local, metapopulation, and cross-community populations differed in their capacity to stabilize metacommunities (Fig. 2A). Cross-community stabilization dominated and accounted for 60.9% ± 19.0 of total stabilization. Mean β_cc_ exceeded that of both β_mp_ and δ *δ*_*2,18*_ = 6.13, *P* = 0.009). β_cc_ involved the largest number of population pairs and hence covariances, followed by δ and β_mp_ (Fig. 2B). δ stabilized little because, even though each local community was strongly stabilizing, there were relatively few local communities in metacommunities (Fig. 2C, D). β_mp_ was similarly small because there were few metapopulations and each one contributed to little stabilization. β_cc_, on the other hand, was large because metacommunities contained many pairs of local communities—which give rise to cross-community population pairs—and each of these strongly stabilized the metacommunity (Fig. 2C, D). Cross-community pairs stabilized the metacommunity more effectively than local or metapopulation pairs due to their higher asynchrony (Fig. 3A; *F*_2,18_ = 12.81, *P* < 0.001) and lower mean correlation (Fig. 3B; *F*_2,_18 = 6.96, *P* = 0.006). We did not detect any differences in the relative abundances or variability of population pairs.

Forms of stabilization had different environmental drivers. δ, for instance, correlated positively with mean dissolved oxygen of rock pools in a metacommunity (*r* = 0.76, *t*_2,5_ = 2.64, P = 0.046). Further analysis showed this was because dissolved oxygen or associated, unmeasured variables acted to decorrelate species and stabilize local communities (Appendix S7: Tables S1, S2). β_mp_ was positively associated with mean salinity (*r* = 0.84, *t*_2,5_ = 3.41, *P* = 0.019), a factor that increased the stabilizing effect of relative abundance on metapopulations. *β*_cc_, on the other hand, declined with local invertebrate abundance (*r* = –0.84, *t*_2,5_ = 3.52, *P* = 0.017), a result of weaker stabilization from relative abundance and variability patterns in high-density pools.

### Diversity-asynchrony relationships

**Fig. 4:**
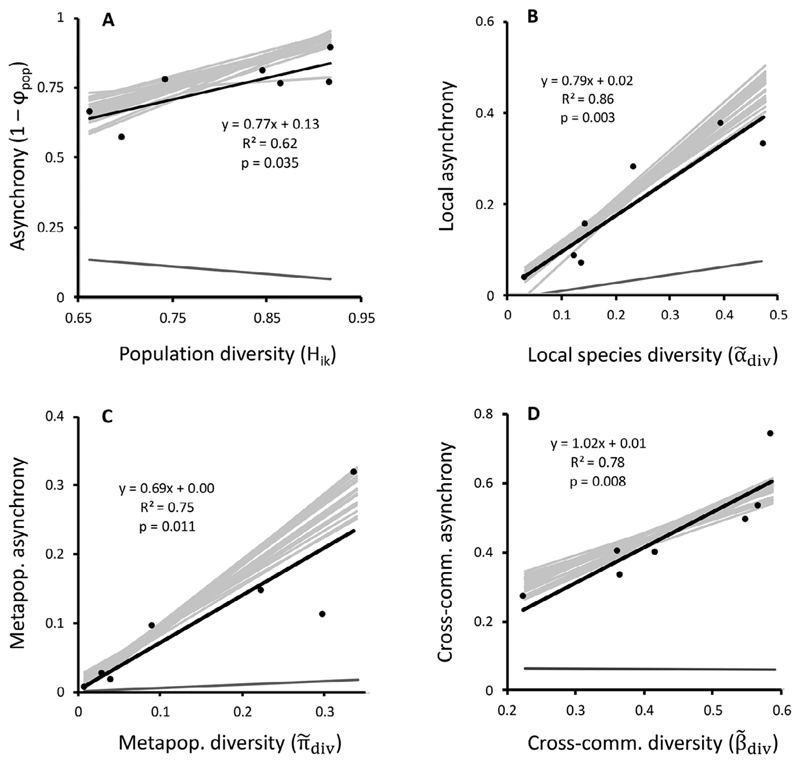
Diversity–asynchrony relationships. (A) Population asynchrony (1 – φ_pop_) increased with Gini-Simpson population diversity. Components of population diversity (Table 2) predicted different types of asynchrony with: (B) local diversity predicting the asynchrony contributed by local communities; (C) diversity of populations in metapopulations predicting the metapopulation fraction of asynchrony; and (D) cross-community diversity predicting cross-community asynchrony. Accompanying lines represent slopes from null cases of uncorrelated populations (light gray lines from 25 permutations) and perfectly correlated populations (dark gray line), generated by data shuffling (see Methods). Local, metapopulation, and cross-community components of asynchrony are defined in Eq. 4.

Population diversity did not predict stabilization (ω instead was a function of population variability, the third factor determining gamma variability in Eq. B2; *R* ^2^ = 0.83, *F*_1,6_ = 28.22, P = 0.002). Rather, diversity was a strong and positive predictor of asynchrony, consistent with Eq. B3 (Fig. 4A). Furthermore, the local, metapopulation, and cross-community components of this diversity (Table 2) predicted asynchrony coming from local, metapopulation, and cross-community sources, respectively (Fig. 4B-D). These diversity-asynchrony relationships were similar in slope and intercept to a null model of weakly correlated populations, created by data randomization (Fig. 4, gray lines). Further null models showed these relationships to be robust to a wide range of population correlation scenarios (Appendix S8: Fig. S1), disappearing only when populations were highly correlated or when metacommunities had very different values of interpopulation correlation (Appendix S8: Fig. S1).

## Discussion

We presented a solution for partitioning forms of asynchrony that are partially or wholly hidden when metacommunities are analyzed as a hierarchy of local communities or metapopulations. By taking a metacommunity as a set of asynchronous local populations, our analytical framework reveals how metacommunities are stabilized by one local and two regional forms of asynchrony (local, metapopulation, and cross-community). Not only is this perspective consistent with the classical conception of metacommunity dynamics (see Introduction; Holyoak et al. 2005:9), but it also unifies the local community and metapopulation approaches to studying metacommunities by capturing stabilization from each (see also Wang et al. 2019). Our empirical results further underscore how diversity and environmental variation support the wide range of stabilizing mechanisms in natural metacom-munities.

### Cross-community stabilization: a hidden source of stability

Cross-community stabilization, while a core of our framework, is seldom recognized as a force smoothing metacommunity variability. This omission is in spite of being deemed necessary for spatial insurance (Gonzalez et al. 2009) and being implicit in finely resolved descriptions of metacommunity dynamics (Gouhier et al. 2010). Yet, this particular form of asynchrony dominated over all others (Fig. 2A), highlighting its importance in reducing variation at the meta-community scale. Since this source of stability is not evident when gamma variability is decomposed as a local community or metapopulation hierarchy (Fig. 1C), studies using these frameworks may underestimate stability arising from asynchrony and miss a unique (spatial 9 species) component of metacommunity dynamics. In turn, recognizing this component will strengthen theoretical and empirical understanding of how spatial heterogeneity and species richness interact to stabilize landscapes, such as through spatial insurance effects.

TTwo factors—one biological and one numeric —may make cross-community stabilization a widespread and potent force in natural ecosystems. First, and biologically, cross-community populations were the least correlated (Fig. 3B) likely due to stronger differential responses to environment. Since Jamaican metacommunities are strongly forced by environmental variation, weak correlation among populations probably owes to differential responses of populations to environmental changes. Thus, the observed weak correlation within local communities (Figs. 2C, 3) is consistent with the local insurance hypothesis (Yachi and Loreau 1999) in which species respond differently to local environmental cues (Leary and Petchey 2009, Thibaut et al. 2012). Weak correlation within metapopulations, in turn, likely reflects the tracking of different environmental regimes by local populations (Ringsby et al. 2002). And the very weak correlations we found among cross-community populations likely stem from differential responses of species across space—the same mechanism of compensatory dynamics in the spatial insurance hypothesis (Loreau et al. 2003). The strength of this stabilizing effect may owe to a doubling up of differential responses: Different species have contrasting responses to the environment and, by living in different local communities, experience different environmental fluctuations. Notably, this effect increased with scale as cross-community asynchrony, pairwise decorrelation and stabilization were accentuated at the whole landscape level (Figs. 2A, 3A, B), presumably and in part as more spatial heterogeneity, and species turnover was included in the sampled area.

Second, the biological causes of high cross-community stabilization are likely to be compounded by the numerical dominance of cross-community populations. We found that the number of cross-community population pairs outstripped the number within local communities or metapopulations (Fig. 2B), with each additional pair adding stabilizing potential akin to a portfolio effect (Blüthgen et al. 2016). Our calculations further suggest that cross-community pairs will dominate in all but the smallest metacommunities (those with less than three local communities and regional species; see Appendix S9: Fig. S1).

We propose that further exploration of the numeric and biological causes of cross-community stabilization will bring important insights about when and where cross-community pairs will contribute most to metacommunity stability. Our framework may also be profitably extended to include functional groups and their specific contributions to stabilization in the local, metapopulation, and cross-community context. We further note that asynchrony specific to ecologically important interactions—such as between predator and prey or plants and pollinators—may also be obscured in current metacommunity frameworks. Future and targeted incorporation of these into partitions will bring ecology closer to a full accounting of stabilizing forces in metacommunities.

### An integrated view of metacommunity stabilization

Our approach allowed for an integrated view of stabilization from local communities and metapopulations. Though cross-community stabilization dominated the metacommunities, stabilization from within local communities and metapopulations was still indispensable and together accounted for nearly half of all stabilization (Fig. 2A). This observation promotes the unifying view that metacommunities are meaningfully stabilized by several lower levels of organization and ecological entities. Thus, sorting out the relative impacts of local communities, metapopulations, and more will be crucial to understanding and managing the stability of landscapes.

With multiple forms of stabilization or asynchrony to balance comes the potential for tradeoffs. Most simplistically, this is because forms of stabilization or asynchrony are collectively exhaustive (Eqs. 2, 4). This property means that given a fixed amount of total stabilization or asynchrony, an increase in one type (e.g., local stabilization) comes at the expense of another (e.g., metapopulation stabilization). Some real-world trade-offs indeed seem possible. Gouhier et al. (2010), for instance, report differential effects of environmental variation on local community and metapopulation asynchrony at certain levels of dispersal. Similarly, managing for one type of asynchrony may unwittingly modify other types. Species-based management, for example, encourages habitat heterogeneity to stabilize metapopulations (e.g., of butterflies; Oliver et al. 2010) but could promote habitats with factors that synchronize local species (e.g., generalist predators; Raimondo et al. 2004). Conversely, community-based management may prioritize species with asynchronous dynamics (e.g., in forests; Morin et al. 2014), but these could include species with easily synchronized local populations (e.g., masting species; Koenig and Knops 2013).

The relative balance of asynchrony forms will also likely be relevant to the maintenance of resilience in metacommunities. Disturbances or management actions that dampen local species asynchrony, for instance, may weaken local insurance effects (Yachi and Loreau 1999). Rescue effects (Brown and Kodric-Brown 1977) similarly depend on metapopulation asynchrony for vigorous local populations to subsidize moribund ones via dispersal. Disruption of cross-community asynchrony, finally, may impair spatial insurance effects (Loreau et al. 2003) in which ecosystem functions are buffered by asynchrony within functional groups (e.g., primary producers; Symons and Arnott 2013). Because rescue, local, and spatial insurance effects depend on different types of asynchrony, an important future research question is how these can be optimized in managed landscapes. Our framework might prove useful for connecting underlying patterns of asynchrony with their associated ecological effects (i.e., rescue, local insurance, and spatial insurance effects).

The multifaceted nature of metacommunity stabilization was also apparent in the variety of environmental controls over stabilization (Appendix S7). Notably, stabilization could be variously promoted or impaired by environmental forcing of the correlation, relative abundance, and variability components of stabilization. If such complex causation is the norm, ecologists will need to move beyond single causes of stabilization (Downing et al. 2014) and elucidate how multiple environmental drivers impact different forms of stabilization and asynchrony. A complete picture of metacommunity stabilization—similar to the local community case (Thibaut and Connolly 2013)—will include understanding how environmental variation differentially affects each statistical component of stabilization (e.g., evenness, correlation, and variability). Absent this detailed understanding, our analysis suggests that preserving biodiversity may be the most viable route to maintaining asynchrony and stability in changing environments (cf. Anderson et al. 2015).

### Multiple paths from diversity to metacommunity stability

Diversity–stability research asks how much and what kind of diversity is needed to support stable ecosystems. Our results show that population diversity increases asynchrony (Fig. 4A), indicating that large metacommunities buffer change in the same way that large financial portfolios enable diversification and variance reduction (Doak et al. 1998, Anderson et al. 2015). Looking deeper, we find that population diversity—and its stabilizing effect—is a composite of other known types of diversity (Eq. B4). Strikingly, at least three diversity–asynchrony relationships stabilize metacommunities and depend on how populations are distributed across local communities and metapopulations.

Alpha diversity, for instance, predicted the amount of asynchrony generated within local communities. This is consistent with previous work showing that a diverse local species pool often buffers community-level variation (Cardinale et al. 2012, Wang and Loreau 2016). Similarly, metapopulation asynchrony grew with the diversity of constituent populations and agreed with studies showing the variance-reducing effects of large metapopulations (Anderson et al. 2015). Cross-community asynchrony, lastly, increased with additive beta diversity. From its equation in Table 2, we see why: As beta diversity grows, so too does the weight of cross-community population pairs and thus their potential contribution to spatial asynchrony. This, combined with the numerical dominance of cross-community pairs, suggests that preserving beta diversity may be of paramount importance for metacommunity stability—a position supported by positive beta diversity–stability relationships in the literature (Mellin et al. 2014, Wang and Loreau 2016).

The diversity–asynchrony relationships we found can be considered portfolio effects because rock pool populations were very weakly correlated (see Figs. 3, 4)—a common assumption of portfolio theory (Doak et al. 1998, Tilman et al. 1998). They may therefore be expected in similarly stochastic metacommunities. But equations and simulations show they may also emerge in more deterministic systems where environmental fluctuations synchronize dynamics. First, Eqs. B1–B3 and our null models predict that the main condition for a positive diversity–asynchrony relationship is simply that an added population has a unique response to environmental fluctuations. Second, diversity–asynchrony relationships are robust to varying levels of interpopulation correlation and only weaken and disappear as populations approach perfect correlation, as predicted by theory (Appendix S8; see also Fig. 5.3 in Loreau 2010). Given this robustness, the smoothing of metacommunity variability by multiple diversity–stability relationships may be a widespread phenomenon. If so, the critical challenge will be to recognize and conserve, not just species diversity (e.g., Leary and Petchey 2009) or patch diversity (Wilcox et al. 2017), but the suite of local community, metapopulation, and cross-community diversities that collectively stabilize landscapes.

## Conclusions

Metacommunity dynamics defy simple analysis and management, at least in part, because they are not tractable by the local community or metapopulation perspective alone. Our novel partition unifies these organizational hierarchies to show how asynchrony arises through multiple local and regional pathways of environmental variation. A more complete view of metacommunity stability will come from recognizing the multiple forms of asynchrony that stabilize metacommunities, gauging their relative importance and studying the diversity-stability relationships which underlie them. We anticipate that highly resolved approaches like ours will prove powerful for disentangling stabilizing mechanisms that span the range of ecological hierarchies (e.g., subpopulations and functional groups).

## Supplementary Material

Additional Supporting Information may be found online at: http://onlinelibrary.wiley.com/doi/10.1002/ecs2.3078/full

Appendix S1

Appendix S2

Appendix S3

Appendix S4

Appendix S5

Appendix S6

Appendix S7

Appendix S8

Appendix S9

## Figures and Tables

**Table 1:** New measures of population-level variability and stabilization by asynchrony in metacommunities (see Appendix S1 for derivations).

Statistic	Measure	Variance-based formula	Coefficient of Variationformula	Related asynchrony measure
ι_CV_	Weighted-average population variability	${\left(\frac{\sum_{ik}\sigma_{ik}}{\sum_{ik}m_{ik}}\right)}^{2}$	(Σ_*ik*_p_*ik*_CV_*ik*_)^2^	-
ω	Stabilization from asynchrony among all local populations	$\frac{\sum_{ik\neq jl}^{jl}\sigma_{ik}\sigma_{jl}-{\text{COV}}_{ik.jl}}{M^{2}}$	$\sum_{ik\neq jl}^{jl}\left(1-\rho_{ik.jl}\right){\tilde{\text{CV}}}_{ik}{\tilde{\text{CV}}}_{jl}$	1 - φ_pop_
δ	Stabilization from asynchrony among species in local communities (type II)	$\frac{\sum_{k}\sum_{i\neq j}^{j}\sigma_{ik}\sigma_{jk}-{\text{COV}}_{ik.jk}}{M^{2}}$	$\sum_{k}\sum_{i\neq j}^{j}\left(1-\rho_{ik.jk}\right)\tilde{{\text{CV}}_{ik}}\tilde{{\text{CV}}_{jk}}$	$\frac{\delta }{\iota_{\text{CV}}}$
β_mp_	Stabilization from asynchrony of populations within metapopulations (type IV)	$\frac{\sum_{i}\sum_{k\neq l}^{l}\sigma_{ik}\sigma_{il}-{\text{COV}}_{ik.il}}{M^{2}}$	$\sum_{i}\sum_{k\neq l}^{l}\left(1-\rho_{ik.il}\right)\tilde{{\text{CV}}_{ik}}\tilde{{\text{CV}}_{il}}$	$\frac{\beta_{\text{mp}}}{\iota_{\text{CV}}}$
B_cc_	Stabilization from asynchrony of different species in different patches (type V)	$\frac{\sum_{k\neq l}^{l}\sum_{i\neq j}^{j}\sigma_{ik}\sigma_{jl}-{\text{COV}}_{ik.jl}}{M^{2}}$	$\sum_{k\neq l}^{l}\sum_{i\neq j}^{j}\left(1-\rho_{ik.jl}\right){\tilde{\text{CV}}}_{ik}{\tilde{\text{CV}}}_{jl}$	$\frac{\beta_{\text{cc}}}{\iota_{\text{CV}}}$

*Notes:* Measures can be expressed using elements of the variance–covariance matrix of metacommunity populations or as products of the relative abundances, temporal CVs, and pairwise correlation coefficients of populations—properties known to influence community-level variability (Cottingham et al. 2001, Thibaut and Connolly 2013). Abbreviations are σ_*ik*_, temporal standard deviation of a population of species *i* in local community *k*; cov_*ik.jl*_, covariance of populations *ik* and *jl*; *m*_*ik*_, temporal mean biomass of population of species *i* in local community *k*; *M*, temporal mean of metacommunity biomass; *CV*, coefficient of variation of a population weighted by its relative abundance in the metacommunity (i.e., *p*_*ik*_CV_*ik*_ where *p*_*ik*_ = *m*_*ik*_/*M*); ρ, between-population Pearson correlation coefficient; φ_pop_, population synchrony index.

**Table 2:** Components of Gini-Simpson diversity (*H*_*ik*_) that increase ω, δ, β_mp_, and β_cc_ stabilization when populations have equal temporal variability and pairwise correlations (see Appendices S4 and S5).

Diversity component	Formula	Probability of sampling two individuals from:	Gini-Simpsondiversity formula	Associated stabilization type
Population	$1-\sum_{ik}p_{ik}^{2}$	Different local populations in the metacommunity	*H*_*ik*_	ω
Local species	$\sum_{k}\sum_{i\neq j}^{j}p_{ik}p_{jk}$	Different species in the same local community	${\tilde{\alpha }}_{\text{div}}=\sum_{k}p_{k}^{2}H_{k}$	δ
Metapopulation	$\sum_{i}\sum_{k\neq l}^{l}p_{ik}p_{il}$	Different populations in the same metapopulation	${\tilde{\pi }}_{\text{div}}=\sum_{i}p_{i}^{2}H_{i}$	β_mp_
Cross-community	$\sum_{k\neq l}^{l}\sum_{i\neq j}^{j}p_{ik}p_{jl}$	Different species from different local communities	${\tilde{\beta }}_{\text{div}}=\gamma_{\text{div}}-{\tilde{\alpha }}_{\text{div}}$	β_cc_

*Notes:* Abbreviations are,*p*_*ik*_ relative abundance of a population, belonging to species *i* and local community *k*, in metacom-munity (i.e., *p*_*ik*_ = m_*ik*_/M); *p*_*k*_ relative abundance of local community *k* in metacommunity (i.e., *p*_*k*_ = m_*k*_/M); p_*i*_, relative abundance of species *i* in metacommunity (i.e., *p*_*k*_ = m_*i*_/M); H_*ik*_ Gini-Simpson diversity index of populations in metacommunity; *H*_*k*_, diversity of species in local community *k*; *H*_*i*_, diversity of populations of species *i*; β _div_, additive beta diversity (Lande 1996); γ_div_ Gini-Simpson species diversity at regional metacommunity scale (i.e., $1-\sum_{i}p_{i}^{2}$). Subscripts are species; *i, j;* local communities; *k, l.*

## Box 1 Linking stabilization and diversity at the metacommunity scale

Diversity–stability relationships are increasingly well understood within local communities in terms of portfolio and insurance effects (McCann 2000, Leary and Petchey 2009, Thibaut and Connolly 2013). But the links between diversity and stability at the metacommunity scale are only now coming into focus (Howeth and Leibold 2010, Wang and Loreau 2016). Analyzing metacommunity stabilization at the resolution of local populations facilitates this effort because many standard diversity measures (e.g., α diversity) also use populations as the basic unit of analysis (e.g., diversity of local species populations).

Viewing the metacommunity as a set of local populations, we can consider the diversity of local populations (i.e., of species i in community k) that contributes to stabilization of total biomass or abundance. Most simply, stabilization always increases with the diversity of local populations when all populations have the same variability (CV) and pairwise correlation (q; Appendix S4):

(B1) $$\omega =H_{ik}\left(1-\rho \right){\text{CV}}^{2}$$

here, *H*_*ik*_ is the Gini-Simpson diversity of all populations in the metacommunity, or $1-\;\sum p_{ik}^{2}$ where *ρ*_ik_ is the relative abundance of a population *ik* in the metacommunity.In real metacommunities where CVs and correlations differ among populations, diversity plays a more contingent role. Here, population diversity (*H*_*ik*_) increases total stabilization unless counteracted by two other factors: population variability (ι_CV_) and asynchrony per unit of diversity (A_H_ which is the ratio of asynchrony to diversity: 1 – φ_pop_ /*H*_*ik*_; see Appendix S4):

(B2) $$\omega \;=\;H_{ik}A_{H}\iota_{\text{CV}}$$

Similar to stabilization, asynchrony increases with diversity unless the added units of diversity are less effective at buffering change and result in a lower asynchrony per unit diversity (i.e., the poor buffering capacity of extra diversity counters the expected gains in asynchrony):

(B3) $$1-\varphi_{\text{pop}}=H_{ik}A_{H}$$

These expressions highlight two routes to stabilizing metacommunity biomass or abundance (cf. Blüthgen et al. 2016), either by increasing population diversity (e.g., portfolio effect) or by increasing asynchrony of that diversity (e.g., increased buffering).

The diversity route to stability depends on the number and evenness of populations in the metacommunity. Usefully, this population diversity can be broken down into contributions from local communities, metapopulations, and cross-community population pairs (Table 2). Similar to species diversity, Gini-Simpson population diversity (H_ik_) is the probability of randomly sampling two individuals from different populations in the metacommunity. These individuals can only be of (1) different species within the same local community, (2) the same species in different local communities, or (3) different species in different local communities. Gini-Simpson diversity in a meta-community thus splits into three components and sampling probabilities reflecting how populations are distributed across species and local communities:

(B4) $$H_{ik}={\tilde{\alpha }}_{\text{div}}+{\tilde{\pi }}_{\text{div}}+{\tilde{\beta }}_{\text{div}}$$

These diversities are implicit in our measures of local, metapopulation, and cross-community stabilization and, in fact, can be derived from them (Appendix S5). These links, in turn, predict four basic diversity–stabilization relationships that will emerge in metacommunities unless obscured by the modifying terms in Eqs. B2, B3 (i.e., diversity that adds extra synchrony or changes population variability):

First, total stabilization of the metacommunity (ω) increases with population diversity (*H*_*ik*_).

Second, local stabilization (d) rises with a measure of local species diversity, ${\tilde{\alpha }}_{\text{div}}$, which is a weighted alpha diversity emphasizing larger local communities (Table 2).

Third, metapopulation stabilization (β_mp_) increases with the diversity of constituent populations, ${\tilde{\pi }}_{\text{div}}$. The same general form ${\tilde{\alpha }}_{\text{div}}{,\tilde{\pi }}_{\text{div}}$, is an average population diversity of metapopulations and is weighted toward dominant species.

Finally, cross-community stabilization (β_cc_) increases with ${\tilde{\beta }}_{\text{div}}$, a weighted, additive beta diversity (Lande 1996). It represents the average amount of diversity not found in a random local community (Veech et al. 2002) with down-weighting of larger local communities.
