# Supplementary material for: Disentangling local, metapopulation, and cross-community sources of stabilization and asynchrony in metacommunities
Source: Ecosphere. Author manuscript; Available in PMC 2020 Dec 14. (PMC7116476; doi:10.1002/ecs2.3078)
Supplement: Appendix S1 [file EMS106906-supplement-Appendix_S1.pdf]

Appendix S1. Derivation of analytical partitions of stabilization

For article: Disentangling local, metapopulation and cross-community sources of stabilization and asynchrony in metacommunities
Journal: Ecosphere
Authors: Matthew Hammond, Michel Loreau, Claire de Mazancourt & Jurek Kolasa

Here we use an approach that parallels Wang and Loreau (2014) to partition temporal variability at the metacommunity scale (gamma variability or γ_{CV}). Wang and Loreau additively partition gamma variability into two components: α_{CV} which represents the (squared) weighted-mean variability of local communities and β which is the amount that asynchrony of local communities reduces γ_{CV}. We also partition γ_{CV} but into the weighted-mean variability of populations in the metacommunity and the variability-reducing effect of asynchrony between those populations. Derivations are reported below both in terms of variances/covariances and Coefficients of Variation (CVs).

a. Derivation of ι_{CV} and ω
Gamma variability is defined as the squared CV of metacommunity biomass or other aggregated quantity (Wang and Loreau, 2014):

$$\gamma_{CV} = CV_M^2 = \frac{Var_M}{M^2} \qquad \text{Eq. S1}$$

where Var_M and *M* are the temporal variance and mean of metacommunity biomass, respectively. Importantly, metacommunity biomass can be obtained by summing local communities k and l, as by Wang and Loreau, or by summing all local populations that occur in the metacommunity, that is, populations of species i in local community k.

Defining metacommunity biomass so lets us additively partition, not the variability of local communities and between-community asynchrony, but the variability of local populations (ι_{CV}) and the stabilization from asynchrony between populations (ω):

$$\gamma_{CV} = \iota_{CV} - \omega \qquad \text{Eq. S2}$$

ι_{CV} is of the same form as Wang and Loreau's (2014) α_{CV} but is applied at a finer resolution in the metacommunity – at the population, not the community, level. It is therefore a weighted-average variability of all populations in the metacommunity:

$$\iota_{CV} = \widetilde{\widetilde{CV}}_{ik}^2 \qquad \text{Eq. S3}$$

where:

$$\widetilde{\widetilde{CV}}_{ik} = \sum_{ik} p_{ik} CV_{ik} \quad \text{Eq. S4}$$

p_{ik} is the relative abundance of a population belonging to species i and local community k in the metacommunity and CV_{ik} is its temporal Coefficient of Variation.

ι_{CV} can also be defined as the value of gamma variability when populations are perfectly synchronized. This equivalence stems from the property that the variability of an aggregate is equal to the average variability of its parts when those parts are perfectly synchronous (Doak et al., 1998). This can be seen by rewriting Eq. S3 in terms of standard deviations and means as:

$$\iota_{CV} = \left(\frac{\sum_{ik} \sigma_{ik}}{\sum_{ik} m_{ik}} \right)^2 \quad \text{Eq. S5}$$

where σ_{ik} and m_{ik} are the temporal standard deviation and mean of a population ik. Expansion of Eq. S5 gives:

$$\iota_{CV} = \frac{\sum_{ik} var_{ik} + \sum_{ik \neq jl}^{jl} \sigma_{ik}\sigma_{jl}}{M^2} \quad \text{Eq. S6}$$

where var_{ik} is the temporal variance of a population of species i in local community k and $\sigma_{ik}\sigma_{jl}$ is the maximum temporal covariance of populations ik and jl which is achieved when they are perfectly synchronized.

Defining ι_{CV} so and rearranging Eq. S2, we can see that the between-population stabilization term (ω) is the difference between observed gamma variability (γ_{CV}) and what gamma variability would be if all populations in the metacommunity were synchronized (ι_{CV}):

$$\omega = \iota_{CV} - \gamma_{CV} \quad \text{Eq. S7}$$

We can further define ω in terms of covariances between populations for later partitioning. As a first step, we express γ_{CV} in variance terms:

$$\gamma_{CV} = \frac{\sum_{ik} var_{ik} + \sum_{ik \neq jl}^{jl} cov_{ik.jl}}{M^2} \quad \text{Eq. S8}$$

where $cov_{ik.jl}$ is the temporal covariance of populations ik and jl.

We then substitute Eqs. S6 and S8 into Eq. S7 and simplify to get:

$$\omega = \frac{\sum_{ik \neq jl}^{jl} \sigma_{ik}\sigma_{jl} - \sum_{ik \neq jl}^{jl} cov_{ik.jl}}{M^2} \quad \text{Eq. S9}$$

Eq. S9 shows ω to be a relativized measure of the difference between perfect and observed covariances between populations, that is a measure of stabilization by asynchrony.

b. Partitioning of ω into δ, β_{mp} and β_{cc}

ω quantifies total stabilization from asynchrony between all pairs of populations in the metacommunity and can be subdivided into further types of stabilization. Note that the numerator of Eq. S9 is the difference of two covariance matrices; one containing the maximum possible covariances between populations in the metacommunity and the other containing the observed covariances. These matrices can be exactly partitioned into covariances between (1) species i and j within local community k, (2) populations of species i inhabiting local communities k and l, and (3) cross-community pairs, that is species i in local community k with species j in local community l. Fig. S1 shows these covariance groupings in a sample matrix. Splitting the covariance matrices of Eq. S9 in this way partitions ω into local (δ), metapopulation (β_{mp}) and cross-community (β_{cc}) components with the following formulas:

$$\delta = \frac{\sum_k \sum_{i\neq j}^{j} \sigma_{ik}\sigma_{jk} - \mathrm{cov}_{ik.jk}}{M^2} \qquad \text{Eq. S10}$$

$$\beta_{mp} = \frac{\sum_i \sum_{k\neq l}^{l} \sigma_{ik}\sigma_{il} - \mathrm{cov}_{ik.il}}{M^2} \qquad \text{Eq. S11}$$

$$\beta_{cc} = \frac{\sum_{k\neq l}^{l} \sum_{i\neq j}^{j} \sigma_{ik}\sigma_{jl} - \mathrm{cov}_{ik.jl}}{M^2} \qquad \text{Eq. S12}$$

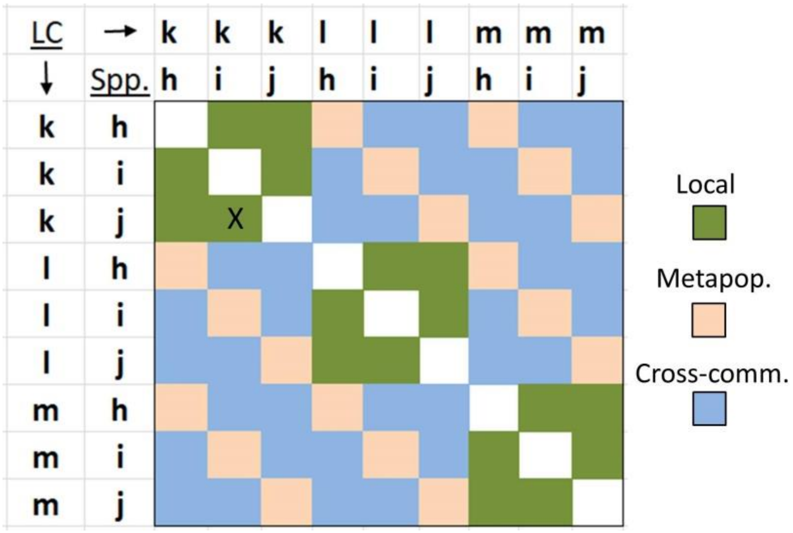

Figure S1. Schematic variance-covariance matrix for calculating stabilization measures. The matrix crosses all populations in the metacommunity, categorized by local community (LC) and species (Spp.). *X*, for example, denotes the temporal covariance of species i in local community k with species j in community k. Three classes of covariances, based on the types of populations crossed, complete the matrix: Local, metapopulation and cross-community.

c. Expressing variance-based metrics in terms of CVs

While γ_{CV} and ι_{CV} are already defined in terms of CVs, metrics of stabilization are not. Here we derive them in terms of the CVs, relative abundances and correlation coefficients of populations. Eq. S9 for total stabilization can be rewritten as:

$$\omega = \frac{\sum_{ik\neq jl}^{jl} \sigma_{ik}\sigma_{jl}}{M^2} - \frac{\sum_{ik\neq jl}^{jl} cov_{ik.jl}}{M^2} \qquad \text{Eq. S13}$$

We next express $cov_{ik.jl}$ as a product of the correlation coefficient $\rho_{ik.jl}$ between populations ik and jl their standard deviations:

$$cov_{ik.jl} = \rho_{ik.jl}\sigma_{ik}\sigma_{jl} \qquad \text{Eq. S14}$$

Substituting this for $cov_{ik.jl}$ in Eq. S13 produces:

$$\omega = \frac{\sum_{ik\neq jl}^{jl} (1 - \rho_{ik.jl})\sigma_{ik}\sigma_{jl}}{M \cdot M} \qquad \text{Eq. S15}$$

Next, we convert metacommunity biomass *M* into expressions of population means m_{ik}. Specifically:

$$M = \frac{m_{ik}}{p_{ik}} \qquad \text{Eq. S16}$$

and

$$M = \frac{m_{jl}}{p_{jl}} \qquad \text{Eq. S17}$$

where p_{ik} and p_{jl} are the relative abundances of populations ik and jl in the metacommunity, respectively. Plugging these identities into Eq. S15, we find:

$$\omega = \sum_{ik\neq jl}^{jl} \frac{(1 - \rho_{ik.jl})p_{ik}p_{jl}\sigma_{ik}\sigma_{jl}}{m_{ik}m_{jl}} \qquad \text{Eq. S18}$$

Quotients of standard deviations and means simplify to the CVs of populations ik and jl as follows:

$$\omega = \sum_{ik\neq jl}^{jl} (1 - \rho_{ik.jl})p_{ik}CV_{ik}p_{jl}CV_{jl} \qquad \text{Eq. S19}$$

Total stabilization is thus expressed in terms of the correlation coefficients and CVs of populations, weighted by their relative abundance in the metacommunity:

$$\omega = \sum_{ik \neq jl}^{jl} (1 - \rho_{ik.jl}) \widetilde{CV}_{ik} \widetilde{CV}_{jl} \qquad \text{Eq. S20}$$

Applying the same approach to Eqs. S10-12 expresses δ, β_{mp} and β_{cc} in terms of the correlation, relative abundance and variability of local, metapopulation and cross-community pairs of populations, as follows:

$$\delta = \sum_{k} \sum_{i \neq j}^{j} (1 - \rho_{ik.jk}) \widetilde{CV}_{ik} \widetilde{CV}_{jk} \qquad \text{Eq. S21}$$

$$\beta_{mp} = \sum_{i} \sum_{k \neq l}^{l} (1 - \rho_{ik.il}) \widetilde{CV}_{ik} \widetilde{CV}_{il} \qquad \text{Eq. S22}$$

$$\beta_{cc} = \sum_{k \neq l}^{l} \sum_{i \neq j}^{j} (1 - \rho_{ik.jl}) \widetilde{CV}_{ik} \widetilde{CV}_{jl} \qquad \text{Eq. S23}$$

Literature cited

Doak, D. F., D. Bigger, E. K. Harding, M. A. Marvier, R. E. O’Malley, and D. Thomson. 1998. The statistical inevitability of stability-diversity relationships in community ecology. The American Naturalist 151:264–276.

Wang, S., and M. Loreau. 2014. Ecosystem stability in space: α, β and γ variability. Ecology letters 17:891–901.
