# Supplementary material for: Disentangling local, metapopulation, and cross-community sources of stabilization and asynchrony in metacommunities
Source: Ecosphere. Author manuscript; Available in PMC 2020 Dec 14. (PMC7116476; doi:10.1002/ecs2.3078)
Supplement: Appendix S2 [file EMS106906-supplement-Appendix_S2.pdf]

Appendix S2. Relating δ, β_{mp} and β_{cc} to existing partitions of metacommunity variability

For article: Disentangling local, metapopulation and cross-community sources of stabilization and asynchrony in metacommunities
Journal: Ecosphere
Authors: Matthew Hammond, Michel Loreau, Claire de Mazancourt & Jurek Kolasa

The main text presents a new partition of metacommunity variability that enables separation of local, metapopulation and cross-community sources of stabilization. This Appendix highlights how our approach differs, conceptually and analytically, from the standard approaches in use. We then relate methods for clarity and future research efforts.

The population-level perspective of metacommunity dynamics
Conceptually, our partition differs from existing one in the scale of ecological dynamics it resolves (Fig. 1; analytical arguments below). Existing "hierarchical" partitions break gamma variability down through an intermediate scale, partitioning along a local community path (metacommunity → local communities → local populations of species), a metapopulation path (metacommunity → metapopulations → local populations) or along both separately (e.g., Wang et al. 2019). Our approach, on the other hand, takes the most finely-resolved unit of the metacommunity - populations of species i in local community k – as the unit of analysis and so omits the intermediate level of hierarchical paths.

In what has become the dominant approach, Wang and Loreau (2014) partition variability along the local community path. This approach aligns well with ecologists' conceptualization of landscapes as nested systems of local communities and resolves stabilization from asynchrony at two scales: The local community (alpha) scale and the scale of species populations within local communities. Resolution at the local community scale reveals *spatial asynchrony among communities* (type I asynchrony) and reflects its coarse-scale drivers like patch disturbance (e.g., McGranahan et al., 2016) and responses to spatial flows. Meanwhile, decomposition to the lower hierarchical level exposes *asynchrony among local species* (type II) and its within-community mechanisms (e.g., competition, differential responses to the local environment). This picture, however, is an incomplete view of population-level dynamics in the metacommunity.

In particular, while we can track spatial asynchrony *among local communities* (a community-level phenomenon), we cannot resolve spatial asynchrony *among members of different local communities* (type IV and V, a population-level phenomenon). Rather, this information is subsumed by the intermediate, local community level. This subsuming renders spatial asynchrony among populations that are of major interest to ecologists – such as metapopulations – undetectable in the local community hierarchy. A similar constraint is faced if the metacommunity is decomposed into a set of metapopulations and then local populations (e.g., Wang et al. 2019). This operation yields *asynchrony among species metapopulations* (type III, a

regional species-level phenomenon) and *asynchrony among populations in metapopulations* (type IV, a population-level phenomenon). But here it is asynchrony of local species (type II) that is subsumed by intermediate levels in the analysis.

Our partitioning retains all population-level information by breaking metacommunity variability down directly to the population scale (Fig. 1, main text). This key step prevents population-level dynamics being obscured by any higher-level dynamics (e.g., local community). Because dynamics are all at the same level they can be broken into local, metapopulation and cross-community subsets that obey the same population-level mechanisms of asynchrony (e.g., differential responses of populations to environment). Moreover, population-level analysis is particularly revealing to ecologists because it is at this scale that (1) well-studied inter- and intra-specific mechanisms operate and (2) classical diversity indices are measured (e.g., alpha diversity of local species populations, population diversity of metapopulations, beta diversity of species populations). The population-level view thus presents new opportunities for interpreting metacommunity dynamics and stability, as we show analytically below.

Analytical comparison of partitions

Partitioning with local communities as the analytical unit

Analytically, Wang and Loreau's (2014) additive approach unfolds over two hierarchical levels and takes local communities as the first grain of analysis:

$$\gamma_{CV} = \alpha_{CV} - \beta \qquad \text{Eq. S1}$$

where γ_{CV} is the variability at the metacommunity scale, α_{CV} is a weighted average variability of local communities and β is the stabilization from asynchrony among local communities (B_2 in Wang and Loreau (2014)). Expanding its formula (Table 1 in Wang and Loreau (2014)), we find that local community variability increases α_{CV} through two terms:

$$\alpha_{CV} = \left(\sum\nolimits_k p_k CV_k\right)^2 = \sum\nolimits_k p_k^2 CV_k^2 + \sum\nolimits_{k\neq l}^{l} p_k CV_k \, p_l CV_l \qquad \text{Eq. S2}$$

where p_k and CV_k are the relative abundance in the metacommunity and Coefficient of Variation of local community k, respectively. The first term reflects variability arising from local communities and the second from pairs (cross-products) of local communities when they are perfectly synchronized. Hierarchical decomposition of the first term then exposes the next hierarchical level and grain of analysis: The variability (CV_k^2) of each local community *k* is broken down in a weighted average variability of local species ($\widetilde{CV}^2_{species}$) and stabilization from asynchrony among local species (which we call $Stab_k$), as $CV_k^2 = \widetilde{CV}^2_{species} - Stab_k$.

Substituting this identity into the first term of Eq. S2, we see that local community variability arises from the variability of local species and stabilization from within-community asynchrony:

$$\sum_k p_k^2 CV_k^2 = \sum_k p_k^2 \widetilde{CV}_{species}^2 - \sum_k p_k^2 \, Stab_k \qquad \text{Eq. S3}$$

Rewriting the second term of Eq. S3 in variance terms, we get:

$$\sum_k p_k^2 \, Stab_k = \frac{\sum_k \sum_{i \neq j}^{j} \sigma_{ik}\sigma_{jk} - cov_{ik.jk}}{M^2} = \delta \qquad \text{Eq. S4}$$

This quantity is a weighted sum of Wang and Loreau's within-community measure, and the amount of stabilization arising from asynchrony within local communities. It is also the same as δ – the local stabilization metric in our framework (see main text, Table 1).

Substituting Eqs. S2-S4 into Eq. S1, we obtain the full hierarchical partition of Wang and Loreau (2014):

$$\gamma_{CV} = \sum_k p_k^2 \widetilde{CV}_{species}^2 + \sum_{k \neq l}^{l} p_k CV_k \, p_l CV_l - \delta - \beta \qquad \text{Eq. S5}$$

Eq. S5 shows sources of metacommunity variability and stabilization spanning two levels: (1) Variability of species within local communities (first term) that is transmitted to the local community scale when species are synchronized; (2) variability of local communities (second term) that is transmitted to the metacommunity scale when they are synchronized; (3) stabilization from species asynchrony within local communities (δ) and (4) stabilization from asynchrony among local communities (β).

Partitioning with species metapopulations as the analytical unit

The second option for a hierarchical decomposition is entirely analogous to the local community partition and proceeds from *metacommunity → metapopulations → local populations* (Wang et al., 2019). The additive partition thus splits gamma variability into S_{CV}, an average variability of species at the metapopulation (regional) scale, and stabilization from asynchrony among those species metapopulations ε.

$$\gamma_{CV} = S_{CV} - \varepsilon \qquad \text{Eq. S6}$$

Similar to the local community partition above, S_{CV} represents the amount of species variability that would propagate to the metacommunity level if all species metapopulations were perfectly synchronized, as follows:

$$S_{CV} = \left(\sum_i p_i CV_i\right)^2 = \sum_i p_i^2 CV_i^2 + \sum_{i \neq j}^{i} p_i CV_i \, p_j CV_j \qquad \text{Eq. S7}$$

We can further express the variability (CV_i^2) of each species metapopulation *i* as a weighted average variability of populations of species i ($\widetilde{CV}_{pop}^2$) and stabilization from asynchrony among populations (which we call $Stab_i$), as $CV_i^2 = \widetilde{CV}_{pop}^2 - Stab_i$. Substituting into Eq. S7, we confirm

that metapopulation variability arises from the variability of local populations and stabilization from asynchrony among those populations:

$$\sum_{i} p_i^2 CV_i^2 = \sum_{i} p_i^2 \widetilde{CV}_{pop}^2 - \sum_{i} p_i^2 \, Stab_i \qquad \text{Eq. S8}$$

Rewriting the stabilizing term of Eq. S8 in variance terms, we show that the within-metapopulation stabilization of the metapopulation hierarchy is the same as β_{mp} in our partition (Table 1):

$$\sum_{i} p_i^2 \, Stab_i = \frac{\sum_i \sum_{k \neq l}^{l} \sigma_{ik}\sigma_{il} - cov_{ik.il}}{M^2} = \beta_{mp} \qquad \text{Eq. S9}$$

The full additive partition based on the metapopulation hierarchy is therefore:

$$\gamma_{CV} = \sum_{i} p_i^2 \widetilde{CV}_{pop}^2 + \sum_{i \neq j}^{j} p_i CV_i \, p_j CV_j - \beta_{mp} - \varepsilon \qquad \text{Eq. S10}$$

Partitioning with local populations as the analytical unit

The partition we present proceeds from *metacommunity → local populations*, with all terms referring to the population level (cf. Eqs. S5, S10): Metacommunity variability arises from the variability of populations in the metacommunity (ι_{CV}, see Eqs. S3-4) and is stabilized by asynchrony among those populations (ω):

$$\gamma_{CV} = \iota_{CV} - \omega \qquad \text{Eq. S11}$$

Subsequent breakdown of ω (Appendix S1: Eqs. S10-S12) partitions stabilization over local and regional scales (i.e., δ within local communities, $\beta_{mp}$and β_{cc} across them), but all types of stabilization still take place between populations (local, metapopulation and cross-community, respectively). This distinction is critical because only at the population level do the community hierarchy (metacommunity → local communities → local populations) and the metapopulation hierarchy (metacommunity → metapopulations → local populations) share the same unit of analysis. This common currency enables two breakthroughs. First, it allows the simultaneous quantification of stabilizing elements from both hierarchies (δ and β_{mp}, see above). Second, analysis at the population level reveals information that is hidden when a hierarchical decomposition with an intermediate level is used.

As an example of the latter, spatial asynchrony of local communities will often mask spatial asynchrony of populations because aggregates (e.g., local communities) subsume lower-level information. Fig. S1 shows that two local communities can be strongly synchronized (β near zero) even while metapopulations exhibit strong asynchrony (high β_{mp}) and cross-community pairs show meaningful spatial asynchrony ($\beta_{cc} > \beta$). In simulated random data, β_{mp} and β_{cc} together are about double β, reinforcing the notion that aggregating to the local community scale obscures spatial asynchrony at the population-level (i.e., among pairs of populations). Thus,

understanding the importance of metapopulation and cross-community pairs of species for metacommunity stability requires a strictly population-level approach.

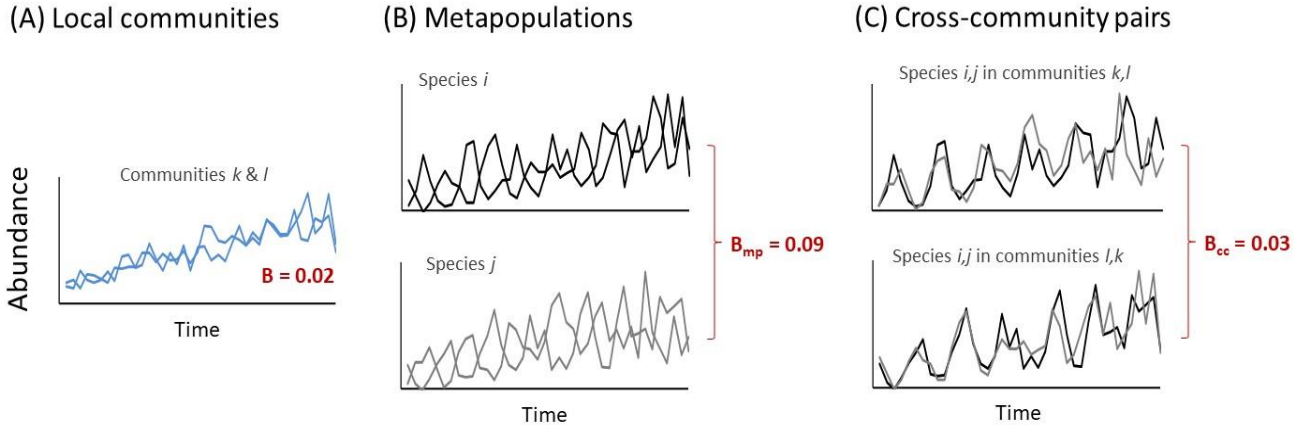

Figure S1. Differences in community- and population-level spatial asynchrony. Local communities can exhibit low asynchrony (A) even while members of those local communities, which comprise metapopulations and cross-community populations, have higher spatial asynchrony (B, C). Note beta values in red.

Cross-scale relationships among partitions

Hierarchical and population-level partitions are complementary, addressing the community-level and population-level mechanisms of metacommunity stability, respectively. To emphasize differences and foster this complementarity, we present cross-scale relationships between our partition and the popular local community partition (Wang and Loreau, 2014).

We begin with the difference between local community-level and population-level stabilization:

$$\omega - \beta = \delta + \beta_{mp} + \beta_{cc} - \beta \qquad \text{Eq. S12}$$

We define the last three terms of Eq. S12 as $\beta^* = (\beta_{mp} + \beta_{cc}) - \beta$. It is the difference in stabilization from spatial asynchrony of local populations (i.e., of metapopulations and cross-community populations) and stabilization from local communities, and so links spatial stabilization at local community and population scales. Specifically, it is the stabilizing by metapopulations and cross-community populations that does not contribute to higher-level stabilization between local communities (e.g., Fig. S1). β^* is zero when all populations in the metacommunity are synchronized (β_{mp}, β_{cc} and β are all zero) and increases as the biomasses of two communities become spatially-synchronized more so than their community members (e.g., two aggrading forests with differing embedded community dynamics).

Inserting β^* into Eq. S12 and rearranging, we show total stabilization ω to be a result of local stabilization (δ), stabilization among local communities (Wang and Loreau's β), and any additional population-level stabilization from metapopulation and metacommunity asynchrony (β^*):

$$\omega = \delta + \beta + \beta^* \quad \text{Eq. S13}$$

Further, rearranging the definition of β^* gives stabilization between local communities (β) as metapopulation and cross-community stabilization ($\beta_{\mathrm{mp}}, \beta_{\mathrm{cc}}$) less spatial stabilization that is purely population-level (β^*) i.e., does not contribute to stabilization at the local community scale:

$$\beta = \beta_{\mathrm{mp}} + \beta_{\mathrm{cc}} - \beta^* \quad \text{Eq. S14}$$

Through the above expressions, we can relate community- and population-level partitions in a hybrid statistical model. In it, metacommunity variability is a function of average population variability (ι_{CV}), stabilized by local species asynchrony (δ), asynchrony among local communities (β), and any additional population-level spatial asynchrony from metapopulations and cross-community populations (β^*):

$$\gamma_{\mathrm{CV}} = \iota_{\mathrm{CV}} - \delta - \beta - \beta^* \quad \text{Eq. S15}$$

Linking functions such as Eq. S15 build a bridge between hierarchical and population-level methods and may be useful for understanding how population-level and community-level mechanisms interact to stabilize metacommunities.

Literature cited

McGranahan, D. A., T. J. Hovick, R. D. Elmore, D. M. Engle, S. D. Fuhlendorf, S. L. Winter, J. R. Miller, and D. M. Debinski. 2016. Temporal variability in aboveground plant biomass decreases as spatial variability increases. Ecology 97:555–560.

Wang, S., and M. Loreau. 2014. Ecosystem stability in space: α, β and γ variability. Ecology letters 17:891–901.

Wang, S., T. Lamy, L. M. Hallett, and M. Loreau. 2019. Stability and synchrony across ecological hierarchies in heterogeneous metacommunities: Linking theory to data. Ecography 42:1200–1211.
