# Supplementary material for: Disentangling local, metapopulation, and cross-community sources of stabilization and asynchrony in metacommunities
Source: Ecosphere. Author manuscript; Available in PMC 2020 Dec 14. (PMC7116476; doi:10.1002/ecs2.3078)
Supplement: Appendix S3 [file EMS106906-supplement-Appendix_S3.pdf]

Appendix S3. Linking stabilization and asynchrony by relating additive and multiplicative partitions

For article: Disentangling local, metapopulation and cross-community sources of stabilization and asynchrony in metacommunities
Journal: Ecosphere
Authors: Matthew Hammond, Michel Loreau, Claire de Mazancourt & Jurek Kolasa

The partitioning of gamma variability into average population variability (ι_{CV}) and total stabilization (ω) is an additive approach that can easily be linked to multiplicative approaches that use Loreau and de Mazancourt's (2008) φ measure of synchrony. To make this link, we first define φ as a measure of synchrony between population ik and jk in a metacommunity:

$$\varphi_{pop} = \frac{Var_M}{\left(\sum_{ik} \sigma_{ik}\right)^2} \qquad \text{Eq. S1}$$

Which, according to Eq. S6 and S8 in Appendix S1, can also be written as:

$$\varphi_{pop} = \frac{\gamma_{CV}}{\iota_{CV}} \qquad \text{Eq. S2}$$

We now convert φ_{pop} into a measure of asynchrony, 1 - φ_{pop}, which yields the following:

$$1 - \varphi_{pop} = 1 - \frac{\gamma_{CV}}{\iota_{CV}} \qquad \text{Eq. S3}$$

Eq. S3 can be rewritten as:

$$1 - \varphi_{pop} = \frac{\iota_{CV} - \gamma_{CV}}{\iota_{CV}} \qquad \text{Eq. S4}$$

The numerator of Eq. S4 is the definition of total stabilization ω (Appendix S1: Eq. S7). Plugging it in, we see that population asynchrony is a ratio of stabilization (reduction of variability) to population variability:

$$1 - \varphi_{pop} = \frac{\omega}{\iota_{CV}} \qquad \text{Eq. S5}$$

Rearranging Eq. S5 for ω and substituting it into Eq. S2 in Appendix S1 gives Eq. 2 of the main text and illustrates how population asynchrony stabilizes gamma variability:

$$\gamma_{CV} = \iota_{CV} - \left(1 - \varphi_{pop}\right)\iota_{CV} \qquad \text{Eq. S6}$$

With the bridge between additive and multiplicative partitions in place, we can now subdivide asynchrony in the same way as we did stabilization. Specifically, we insert the additive partition $\omega = \delta + \beta_{mp} + \beta_{cc}$ (Eq. 3 in main text) into the numerator of Eq. S5 to give:

$$1 - \varphi_{pop} = \frac{\delta + \beta_{mp} + \beta_{cc}}{\iota_{CV}} = \frac{\delta}{\iota_{CV}} + \frac{\beta_{mp}}{\iota_{CV}} + \frac{\beta_{cc}}{\iota_{CV}} \qquad \text{Eq. S7}$$

The result is three fractions of population asynchrony that correspond (from left to right) to asynchrony generated by local, metapopulation and cross-community pairs of populations.

Literature cited

Loreau, M., and C. de Mazancourt. 2008. Species synchrony and its drivers: Neutral and nonneutral community dynamics in fluctuating environments. The American Naturalist 172: E48–E66.
