# Supplementary material for: Disentangling local, metapopulation, and cross-community sources of stabilization and asynchrony in metacommunities
Source: Ecosphere. Author manuscript; Available in PMC 2020 Dec 14. (PMC7116476; doi:10.1002/ecs2.3078)
Supplement: Appendix S4 [file EMS106906-supplement-Appendix_S4.pdf]

Appendix S4. Linking diversity and stabilization

For article: Disentangling local, metapopulation and cross-community sources of stabilization and asynchrony in metacommunities
Journal: Ecosphere
Authors: Matthew Hammond, Michel Loreau, Claire de Mazancourt & Jurek Kolasa

Eq. S19 in Appendix S1 shows that total stabilization (ω) depends on the correlation and variability of populations, as well as the relative abundance structure of the underlying communities. In this Appendix, we derive the relationship between stabilization or asynchrony and common indices of diversity, both with and without simplifying assumptions.

We first assume that all populations in the metacommunity have the same CV (CV_{ik}) and the same pairwise correlation coefficient ($\rho_{ik.jl}$). Doing so allows us to move these elements outside of the summation and rewrite Eq. S19 (Appendix S1) as:

$$\omega = \left(1 - \rho_{ik.jl}\right)CV_{ik}^2 \cdot \sum_{ik \neq jl}^{jl} p_{ik}p_{jl} \qquad \text{Eq. S1}$$

Stabilization of a simplified metacommunity thus depends on the correlation, variability and relative abundance structure of populations. The latter is expressed in terms of $p_{ik}p_{jl}$ which is the cross product of the relative abundance of population ik and jl in the metacommunity (i.e., m_{ik}/M and m_{jl}/M, respectively).

We note that $\sum_{ik \neq jl}^{jl} p_{ik}p_{jl}$ in Eq. S1 is the Gini-Simpson Index of the diversity of populations in the metacommunity, which would usually be written as $1 - \sum_{ik} p_{ik}^2$. Denoting this index H_{ik}, we can now show that – all else equal – total stabilization increases with population diversity:

$$\omega = H_{ik}\,(1 - \rho)\,CV^2 \qquad \text{Eq. S2}$$

Next, we relax the above assumptions that all populations have the same CV and pairwise correlation to obtain a diversity-stabilization function that holds under all conditions. We begin by noting from Eq. S5 in Appendix S3 that:

$$\omega = \left(1 - \varphi_{pop}\right)\iota_{CV} \qquad \text{Eq. S3}$$

We then insert a partition that casts total stabilization in terms of H_{ik}, ι_{CV} and a simple ratio of population asynchrony to diversity:

$$\omega = H_{ik} \cdot \iota_{CV} \cdot \frac{1 - \varphi_{pop}}{H_{ik}} \qquad \text{Eq. S4}$$

We denote the ratio of $1 - \varphi_{pop}$ to H_{ik} as A_H to represent the amount of asynchrony per unit of Gini-Simpson diversity:

$$A_H = \frac{1 - \varphi_{pop}}{H_{ik}} \qquad \text{Eq. S5}$$

A_H has a minimum value of 0 when all populations contributing to diversity are perfectly synchronized. It reaches its maximum value of $1/H_{ik}$ when perfect asynchrony reduces gamma variability to zero. We note that this maximum per capita asynchrony declines with diversity, likely reflecting the known constraint that more than two populations cannot be perfectly negatively correlated (Loreau and de Mazancourt, 2008). The necessity that some populations fall short of perfect negative correlation translates into less asynchrony per unit of diversity as population diversity grows. This interpretation is consistent with observations that asynchrony saturates with diversity (e.g., Doak et al., 1998), implying that each additional unit of diversity tends to contribute less to asynchrony. Viewed another way, all else equal, removing a population would severely impact asynchrony in a system of two populations, but would have a negligible effect in a system of 100 populations. Thus, asynchrony per unit of diversity should decline with diversity.

Literature cited

Doak, D. F., D. Bigger, E. K. Harding, M. A. Marvier, R. E. O'Malley, and D. Thomson. 1998. The statistical inevitability of stability-diversity relationships in community ecology. The American Naturalist 151:264–276.

Loreau, M., and C. de Mazancourt. 2008. Species synchrony and its drivers: Neutral and nonneutral community dynamics in fluctuating environments. The American Naturalist 172: E48–E66.
