# Supplementary material for: Disentangling local, metapopulation, and cross-community sources of stabilization and asynchrony in metacommunities
Source: Ecosphere. Author manuscript; Available in PMC 2020 Dec 14. (PMC7116476; doi:10.1002/ecs2.3078)
Supplement: Appendix S5 [file EMS106906-supplement-Appendix_S5.pdf]

Appendix S5. Deriving diversity components for different forms of stabilization

For article: Disentangling local, metapopulation and cross-community sources of stabilization and asynchrony in metacommunities
Journal: Ecosphere
Authors: Matthew Hammond, Michel Loreau, Claire de Mazancourt & Jurek Kolasa

In Appendix S4, we linked total stabilization to the Gini-Simpson Index of population diversity, which is interpreted as the probability of drawing two individuals from different populations with random sampling. The same derivation can be applied to other types of stabilization. Applying assumptions of equal correlation and population variability to the formulas for δ, β_{mp} and β_{cc} (Appendix S1: Eqs. S21-23) yields:

$$\delta = (1 - \rho_{ik.jl})CV_{ik}^2 \cdot \sum_k \sum_{i \neq j}^{j} p_{ik}p_{jk} \qquad \text{Eq. S1}$$

$$\beta_{mp} = (1 - \rho_{ik.jl})CV_{ik}^2 \cdot \sum_i \sum_{k \neq l}^{l} p_{ik}p_{il} \qquad \text{Eq. S2}$$

$$\beta_{cc} = (1 - \rho_{ik.jl})CV_{ik}^2 \cdot \sum_{k \neq l}^{l} \sum_{i \neq j}^{j} p_{ik}p_{jl} \qquad \text{Eq. S3}$$

By their definitions, δ, β_{mp} and β_{cc} sum to ω. Summing Eqs. S1-3 and simplifying thus gives:

$$\omega = (1 - \rho_{ik.jl})CV_{ik}^2 \cdot \left(\sum_k \sum_{i \neq j}^{j} p_{ik}p_{jk} + \sum_i \sum_{k \neq l}^{l} p_{ik}p_{il} + \sum_{k \neq l}^{l} \sum_{i \neq j}^{j} p_{ik}p_{jl}\right) \qquad \text{Eq. S4}$$

Substituting Eq. S4 into Eq. S4 in Appendix S4 and simplifying shows that population diversity H_{ik}, which promotes total stabilization, can be divided into three components that promote δ, β_{mp} and β_{cc} (Eqs. S1-S3):

$$H_{ik} = \sum_k \sum_{i \neq j}^{j} p_{ik}p_{jk} + \sum_i \sum_{k \neq l}^{l} p_{ik}p_{il} + \sum_{k \neq l}^{l} \sum_{i \neq j}^{j} p_{ik}p_{jl} \qquad \text{Eq. S5}$$

Note that while we make assumptions to derive diversity components from δ, β_{mp} and β_{cc}, the components can be also be derived without these assumptions by directly partitioning H_{ik} itself (see Eq. 8 in main text). The first component and term of Eq. S5 corresponds to a *local diversity* component of H_{ik}, that is the probability of sampling different species (i and j) from the same local community (k) based on their relative abundance in the metacommunity. The second term gives a *metapopulation diversity* - the probability of sampling two populations (in local communities k and l) of the same species i. The last term gives a *cross-community diversity* which is the probability of sampling two populations of different species (i and j) from different local communities (k and l).

To facilitate interpretation, we next express the probabilistic diversity components in Eq. S5 as Gini-Simpson diversity indices. We begin with the local diversity component $\sum_k \sum_{i \neq j}^{j} p_{ik}p_{jk}$,

noting that p_{ik} – the relative abundance of population *ik* in the metacommunity – can be rewritten as the product of two relative abundances:

$$\mathbf{p_{ik} = p_k p_{i\_k}} \qquad \text{Eq. S6}$$

where p_k is the relative abundance of local community k in the metacommunity (i.e., m_k/M) and p_{i_k} is the relative abundance of species i in local community k (i.e., m_{ik}/m_k). Applying this identity, the local diversity component becomes:

$$\sum_k p_k^2 \sum_i^j p_{i\_k} p_{j\_k} \qquad \text{Eq. S7}$$

The term $\sum_i^j p_{i\_k} p_{j\_k}$ can be rewritten as $\sum_i \ 1 - p_{i\_k}^2$. This quantity, H_k, is the Gini-Simpson species diversity of local community *k*. Substituting this into Eq. S7 defines the local species diversity component – which we call $\tilde{\alpha}_{div}$ – as:

$$\tilde{\alpha}_{div} = \sum_k p_k^2 H_k \qquad \text{Eq. S8}$$

Metapopulation diversity is derived from the term $\sum_i \sum_{k \neq l}^l p_{ik} p_{il}$ in a similar manner, noting that the relative abundance of population *ik* in the metacommunity can be rewritten as:

$$p_{ik} = p_i p_{k\_i} \qquad \text{Eq. S9}$$

where p_i is the relative abundance of species i in the metacommunity (i.e., m_i/M) and p_{k_i} is the relative abundance of population k in a metapopulation of species i (i.e., m_{ik}/m_i). The metapopulation diversity component becomes:

$$\sum_i p_i^2 \sum_k^l p_{k\_i} p_{l\_i} \qquad \text{Eq. S10}$$

The term $\sum_k^l p_{k\_i} p_{l\_i}$ is equivalent to $\sum_k \ 1 - p_{k\_i}^2$ – the Gini-Simpson diversity of populations in a metapopulation of species i, which we call H_i. Plugging this into Eq. S10 defines the metapopulation component of diversity, $\tilde{\pi}_{div}$, as:

$$\tilde{\pi}_{div} = \sum_i p_i^2 H_i \qquad \text{Eq. S11}$$

Next, we derive the cross-community component of diversity from γ_{div}, the Gini-Simpson index of regional species diversity i.e., $\sum_i \ 1 - p_i^2$. This quantity can also be written as:

$$\gamma_{div} = \sum_{i \neq j}^j p_i \, p_j \qquad \text{Eq. S12}$$

An alternative way of expressing p_i is as a sum of the relative abundances of populations ik and il in the metacommunity, as follows for p_i and p_j:

$$p_i = \sum_k^l p_{ik} \qquad \text{Eq. S13}$$

$$p_j = \sum_k^l p_{jk} \qquad \text{Eq. S14}$$

Substituting Eqs. S13 and S14 into Eq. S12 and expanding the terms yields:

$$\gamma_{div} = \sum_k \sum_{i \neq j}^{j} p_{ik} p_{jk} + \sum_{k \neq l}^{l} \sum_{i \neq j}^{j} p_{ik} p_{jl} \qquad \text{Eq. S15}$$

The left-hand term is the local diversity component, $\tilde{\alpha}_{div}$, while the righthand term is the cross-community component of diversity. Rearranging gives the cross-community term as:

$$\sum_{k \neq l}^{l} \sum_{i \neq j}^{j} p_{ik} p_{jl} = \gamma_{div} - \tilde{\alpha}_{div} \qquad \text{Eq. S16}$$

Cross-community diversity is thus the Gini-Simpson gamma diversity minus a weighted alpha diversity. The difference between gamma and alpha diversity is an additive measure of beta diversity (Lande 1996). Since cross-community diversity is gamma diversity minus a weighted alpha diversity, we consider it a weighted beta diversity, $\tilde{\beta}_{div}$.

$$\tilde{\beta}_{div} = \gamma_{div} - \tilde{\alpha}_{div} \qquad \text{Eq. S17}$$

Eq. S8 shows that $\tilde{\alpha}_{div}$ upweights the diversity of large communities while Eq. S17 shows that $\tilde{\beta}_{div}$ shrinks as $\tilde{\alpha}_{div}$ grows. $\tilde{\beta}_{div}$ is therefore weighted oppositely to $\tilde{\alpha}_{div}$ and downweights large local communities.

Literature cited

Lande, R. 1996. Statistics and partitioning of species diversity and similarity among multiple communities. Oikos 76:5–13.
