# Supplementary material for: Disentangling local, metapopulation, and cross-community sources of stabilization and asynchrony in metacommunities
Source: Ecosphere. Author manuscript; Available in PMC 2020 Dec 14. (PMC7116476; doi:10.1002/ecs2.3078)
Supplement: Appendix S6 [file EMS106906-supplement-Appendix_S6.pdf]

Appendix S6. Comparison of gamma variability to theoretical benchmarks

For article: Disentangling local, metapopulation and cross-community sources of stabilization and asynchrony in metacommunities
Journal: Ecosphere
Authors: Matthew Hammond, Michel Loreau, Claire de Mazancourt & Jurek Kolasa

This Appendix presents results comparing observed gamma variability to benchmarks of theoretical metacommunities with different statistical properties.

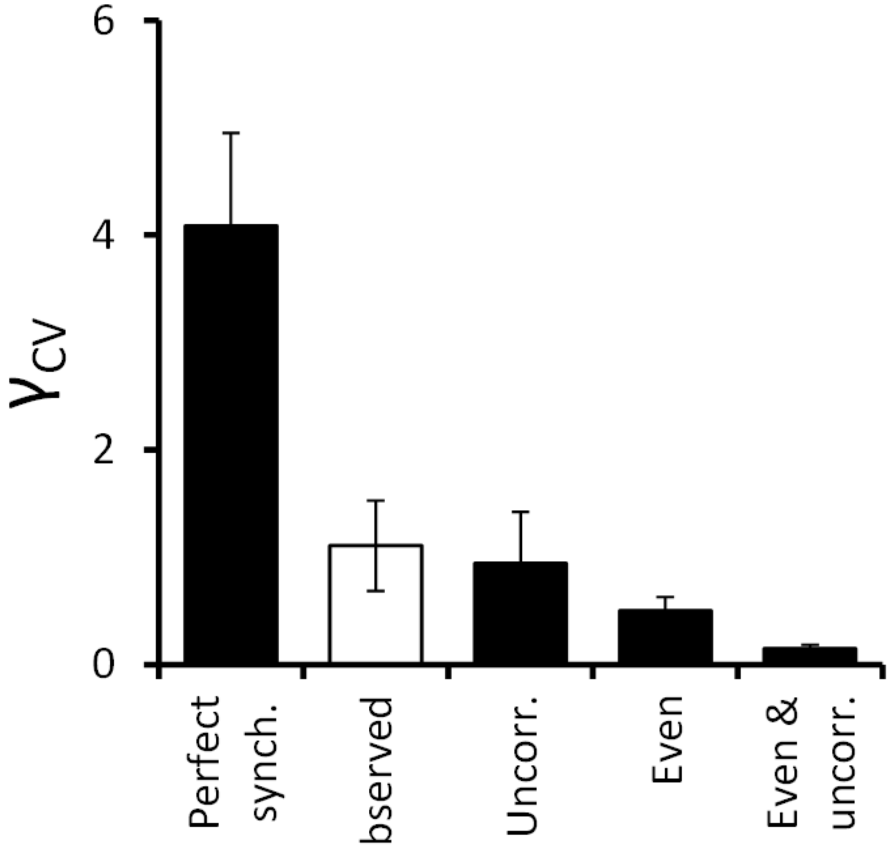

Figure S1. Observed gamma variability (γ_{CV}) of metacommunities compared to theoretical benchmark values for: (i) Perfectly correlated populations ($\rho = 1$; γ_{CV} is equivalent to ι_{CV}), (ii) uncorrelated populations ($\rho = 0$), (iii) even population sizes and (iv) uncorrelated and even populations. Benchmarks are calculated from equations in Table 1 (see Materials & Methods for details).
