# Supplementary material for: Disentangling local, metapopulation, and cross-community sources of stabilization and asynchrony in metacommunities
Source: Ecosphere. Author manuscript; Available in PMC 2020 Dec 14. (PMC7116476; doi:10.1002/ecs2.3078)
Supplement: Appendix S7 [file EMS106906-supplement-Appendix_S7.pdf]

Appendix S7. Environmental drivers of different types of stabilization

For article: Disentangling local, metapopulation and cross-community sources of stabilization and asynchrony in metacommunities
Journal: Ecosphere
Authors: Matthew Hammond, Michel Loreau, Claire de Mazancourt & Jurek Kolasa

δ, β_{mp} and β_{cc} capture stabilization from local, metapopulation and cross-community pairs of populations, respectively. Eqs. S21-23 in Appendix S1 show that they depend on properties of richness, evenness, temporal variability and pairwise correlation of metacommunity components, which is consistent with earlier work (e.g., Tilman 1998; Thibaut & Connolly 2013). Specifically, δ, β_{mp} and β_{cc} are summed products of the relative abundances, temporal CVs and correlation coefficients of populations. There are two ways of evaluating the role these properties play in generating asynchrony and stability. First, a quantity (e.g., cross product of two population CVs) can be averaged across population pairs and these averages used in regressions to find causes. The averaging approach is straightforward for cross-products of two population CVs. The approach is biased, however, for averaging pairwise correlation coefficients (we correct for this bias in one of our analyses; see Materials & Methods). Similarly, the lower bound of mean relative abundance decreases with the number of components averaged, from 1 when there is one component to near-zero when there are many components.

A second approach circumvents the problem of bias by not directly averaging relative abundances or pairwise correlation coefficients. Rather it looks at the contribution of a given property (e.g., relative abundance) makes to stabilizing gamma variability. This is done by comparing observed stabilization to null benchmarks and yields the *stabilizing effect of correlation or relative abundance*, which can then be related to causal factors. We applied this approach to δ, β_{mp} and β_{cc} but use δ below for illustration:

$$\text{Stabilizing effect of (de)correlation} = \frac{\delta}{\delta_{\rho.\emptyset}} \qquad \text{Eq. S1}$$

where δ is observed local stabilization and $\delta_{\rho.\emptyset}$ is the calculated stabilization under the null condition of zero-correlation among populations (i.e., $\rho_{ik.jk} = 0$);

$$\text{Stabilizing effect of relative abundance} = \frac{\delta}{\delta_{e}} \qquad \text{Eq. S2}$$

where δ_e is the stabilization under the null condition of even populations in the metacommunity (i.e., $p_{ik} = 1/N_{pop}$) and;

Ratio values above 1 indicate that a property is responsible for more stabilization than expected under the null condition. These benchmarked contributions have an interpretational advantage over simple means when looking for environmental drivers of stabilization. Mean correlation

(unbiased), for example, may decrease over an environmental gradient but this does not necessarily mean that stabilization does too since this also depends on the relative abundance and variability of components. But an increase in the *stabilizing effect of (de)correlation* unambiguously indicates that decorrelation is responsible for more stabilization at one end of the environmental gradient than the other. We found different positive and negative environmental drivers of stabilizing effects of decorrelation, relative abundance and variability. These are reported with suggested mechanisms in Table S1 along with summary environmental data for the metacommunities in Table S2.

Table S1. Environmental drivers of the decorrelation, relative abundance and variability determinants of stabilization (see equations in Table 1; Eqs. S21-23 in Appendix S1). Decorrelation and relative abundance contributions to stabilization were measured according to Eqs. S1-S2 as ratios of observed stabilization values to select theoretical benchmarks. Variability was compared using means of population CVs.

Stabilizing effect of:	Source	Explanatory variable(s)	r	Interpretation/suggested mechanism
Decorrelation	Local	Pool pH, dissolved oxygen**	0.89	Alkaline pools host more dynamic local communities
$(1 - \rho)$	Metapop.	Number of drying events	-0.66	Droughts synchronize populations
	Cross-comm.	Pool water volume*	0.77	Large pools shelter communities from environmental forcing
Relative abundance	Local	Pool salinity*	0.75	Local marine communities are more even than brackish ones
$(p_{ik}p_{jl})$	Metapop.	Pool salinity*	0.76	Marine generalists (e.g., crab larvae) establish large, even metapopulations
	Cross-comm.	Local community abundance**	-0.89	High-abundance communities are more uneven
Variability	Local	Pool salinity**	0.89	Marine populations are more sensitive to periodic freshwater intrusion
$(CV_{ik}CV_{jl})$	Metapop.	Variability of 1° production*	0.70	Variable primary production destabilizes local populations
	Cross-comm.	Local community abundance*	-0.87	Larger populations are less variable because of reduced demographic stochasticity

Table S2. Environmental parameters of metacommunities in the Jamaican rock pool system. For each environmental driver of stabilization (Table S1), we report mean value and temporal Coefficient of Variation (CV) across the pools.

Variable	Meta-community	Mean	Mean temporal CV
Pool volume (cm^3)	A	10497.45	Insufficient data
	B	15150.84	
	C	17527.40	
	D	15732.14	
	E	15506.41	
	F	12777.32	
	G	29369.25	
Number of drying events	A	2.67	Insufficient data
	B	10.33	
	C	5.00	
	D	3.00	
	E	0.00	
	F	5.00	
	G	3.76	
Temperature (˚C)	A	25.65	0.07
	B	27.52	0.07
	C	27.49	0.09
	D	25.42	0.06
	E	27.08	0.07
	F	26.99	0.09
	G	26.30	0.07
pH	A	8.34	0.06
	B	8.82	0.08
	C	8.89	0.07
	D	8.02	0.04
	E	8.66	0.06
	F	8.56	0.08
	G	8.50	0.07
Salinity (‰)	A	0.48	1.33
	B	1.18	1.04
	C	3.12	0.67
	D	0.62	1.37
	E	15.55	0.77
	F	17.73	0.57
	G	12.96	1.00

Table S2 (cont.)

Variable	Meta-community	Mean	Mean temporal CV
Dissolved oxygen (mg/L)	A	6.55	0.45
	B	7.83	0.37
	C	8.75	0.37
	D	4.18	0.45
	E	8.84	0.34
	F	8.24	0.43
	G	6.62	0.47
Local invertebrate abundance (individuals)	A	675.00	0.96
	B	859.08	1.53
	C	424.10	2.31
	D	942.98	0.92
	E	812.19	1.11
	F	679.56	1.47
	G	637.05	1.21
Primary production (ug/L chlorophyll a)	A	7.12	1.06
	B	7.11	1.31
	C	37.12	1.46
	D	9.49	0.66
	E	35.66	1.25
	F	10.72	1.28
	G	13.75	1.09

Literature cited

Thibaut, L. M., and S. R. Connolly. 2013. Understanding diversity-stability relationships: Towards a unified model of portfolio effects. Ecology Letters 16:140–50.

Tilman, D., C. L. Lehman, and C. E. Bristow. 1998. Diversity-stability relationships: Statistical inevitability or ecological consequence? The American Naturalist 151:277–282.
