# Supplementary material for: Disentangling local, metapopulation, and cross-community sources of stabilization and asynchrony in metacommunities
Source: Ecosphere. Author manuscript; Available in PMC 2020 Dec 14. (PMC7116476; doi:10.1002/ecs2.3078)
Supplement: Appendix S8 [file EMS106906-supplement-Appendix_S8.pdf]

Appendix S8. Null diversity-asynchrony relationships

For article: Disentangling local, metapopulation and cross-community sources of stabilization and asynchrony in metacommunities
Journal: Ecosphere
Authors: Matthew Hammond, Michel Loreau, Claire de Mazancourt & Jurek Kolasa

In this Appendix, we provide further null model simulations to establish the conditions necessary for the emergence of the diversity-asynchrony relationships reported in Fig. 4 of the main text. Fig. S1 compares observed results with null relationships obtained by varying inter-population correlation coefficients in formulas in Table 1 of the main text. Consistent with results of data randomizations (Fig. 4, main text), theoretical relationships show that metacommunities were composed of weakly correlated (i.e., near zero) populations (Fig. S1). Progressively higher correlation had the effect of reducing the slope, and intercept (see Fig. S1a), of diversity-asynchrony relationships.

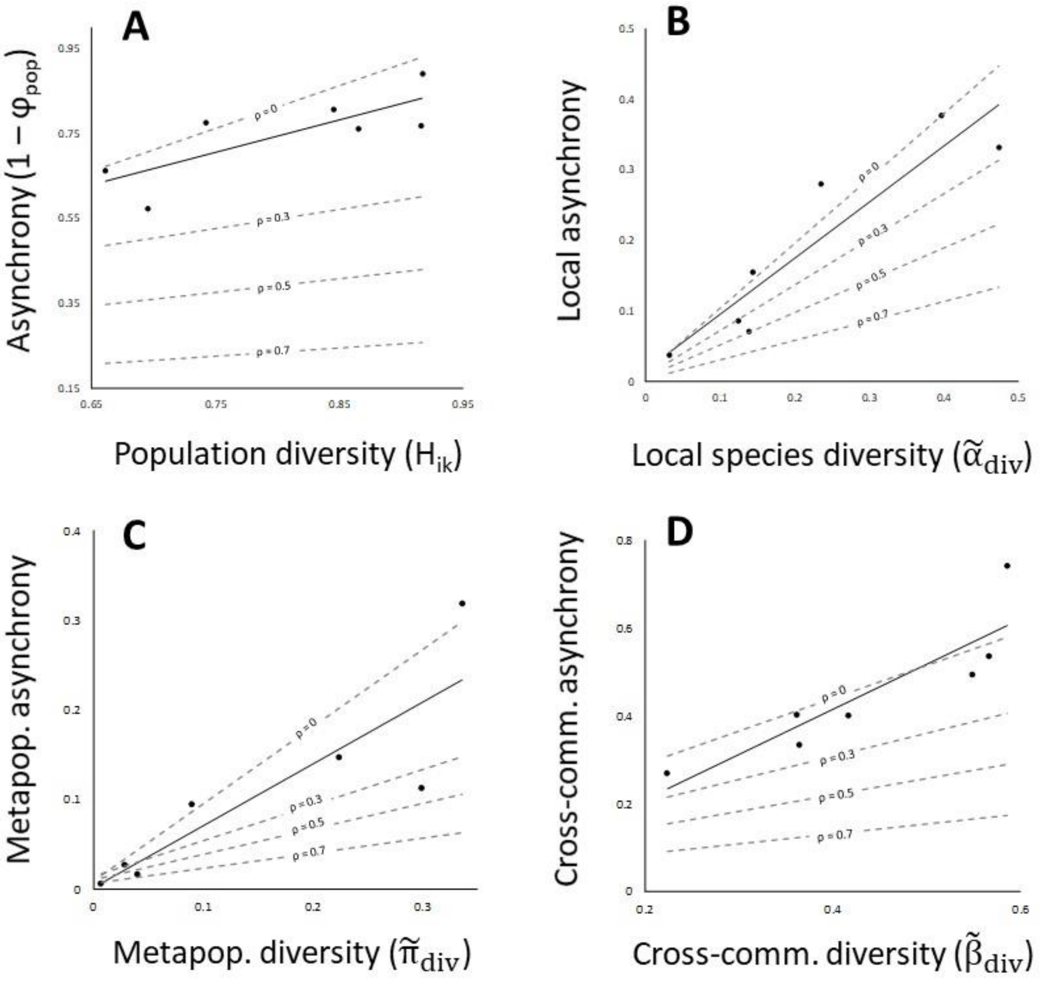

Figure S1. Diversity-asynchrony relationships from Fig. 4 (main text) compared with theoretical relationships obtained by varying inter-population correlation coefficients in Table 1 equations.

Diversity-asynchrony relationships were also weakened by random and large differences in population correlation coefficients among metacommunities (Fig. S2). As the differences in correlation among metacommunities grew, diversity-asynchrony relationships became non-significant, supporting the notion from Eq. 7 (main text) that diversity is stabilizing so long as asynchrony per unit diversity (A_H) is relatively constant.

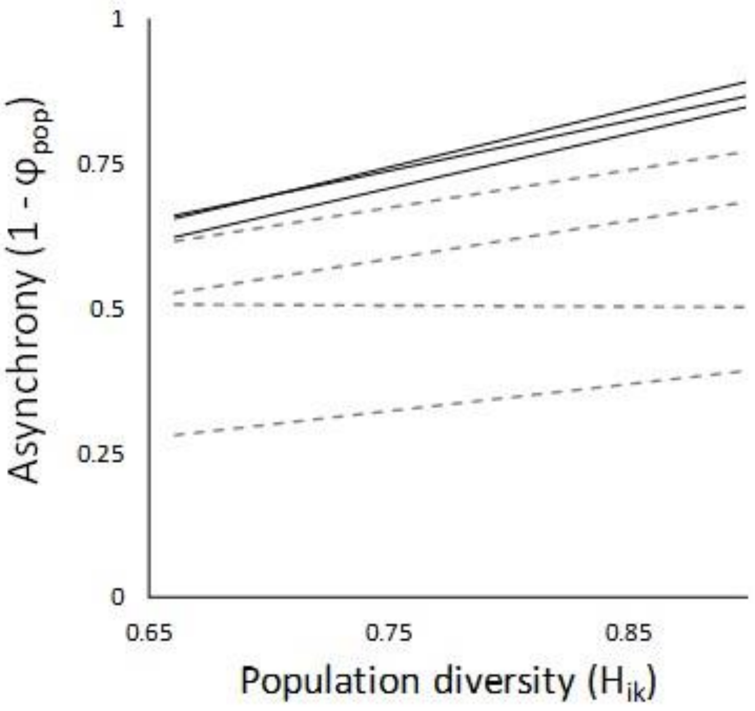

Figure S2. Weakening of diversity-asynchrony slopes with variation in inter-population correlation among metacommunities. Correlation coefficients of populations in metacommunities spanned (from top slopes to bottom) ranges of 0.05, 0.10, 0.15, 0.3, 0.5, 1 and 0.7. Solid lines denote statistically significant slopes.
