# Supplementary material for: Disentangling local, metapopulation, and cross-community sources of stabilization and asynchrony in metacommunities
Source: Ecosphere. Author manuscript; Available in PMC 2020 Dec 14. (PMC7116476; doi:10.1002/ecs2.3078)
Supplement: Appendix S9 [file EMS106906-supplement-Appendix_S9.pdf]

Appendix S9. Expected number of population pairs underlying sources of stabilization

For article: Disentangling local, metapopulation and cross-community sources of stabilization and asynchrony in metacommunities
Journal: Ecosphere
Authors: Matthew Hammond, Michel Loreau, Claire de Mazancourt & Jurek Kolasa

A central consideration in diversity-stability relationships is the number of components that can fluctuate asynchronously. Early work showed that when species could be assumed even in their relative abundance and CVs, asynchrony grew in tandem with the number of uncorrelated species (e.g., Doak et al., 1998; Tilman 1998). All correlations among components are not equal, however. Our work shows that the total asynchrony among populations can be subdivided into local, metapopulation and cross-community pairs. The size of these sources depends on two aspects; the number of population pairs that can add asynchrony and the asynchrony per pair, which is driven by the correlation, relative abundances and CVs of populations.

Here we report the conditions under which δ, β_{mp} or β_{cc} will dominate for reasons of population richness and number of population pairs alone. We therefore ignore any role of correlation, relative abundances and variability in generating asynchrony. The following equations report the number of pairings that are possible for a matrix crossing all populations in metacommunity:

$$N_{loc} = L(S^2 - S) \quad \text{Eq. S1}$$

$$N_{mp} = S(L^2 - L) \quad \text{Eq. S2}$$

$$N_{cc} = (S^2 - S)(L^2 - L) \quad \text{Eq. S3}$$

where N_{loc}, N_{mp} and N_{cc} are the number of pairings of local, metapopulation and cross-community populations, respectively. L is the number of local communities and S is the regional species richness (note that for simplicity we assume that all species are represented in each local community).

Eqs. S1-S3 show numbers of all pairings increasing with both L and S. Notable is that N_{cc} increases with the squares of both S and L, suggesting that cross-community pairs become very numerous and dominate as S and L increase. Fig. S1 confirms that cross-community pairs make up the majority (>50%) of population pairings whenever a metacommunity has more than three local communities and regional species.

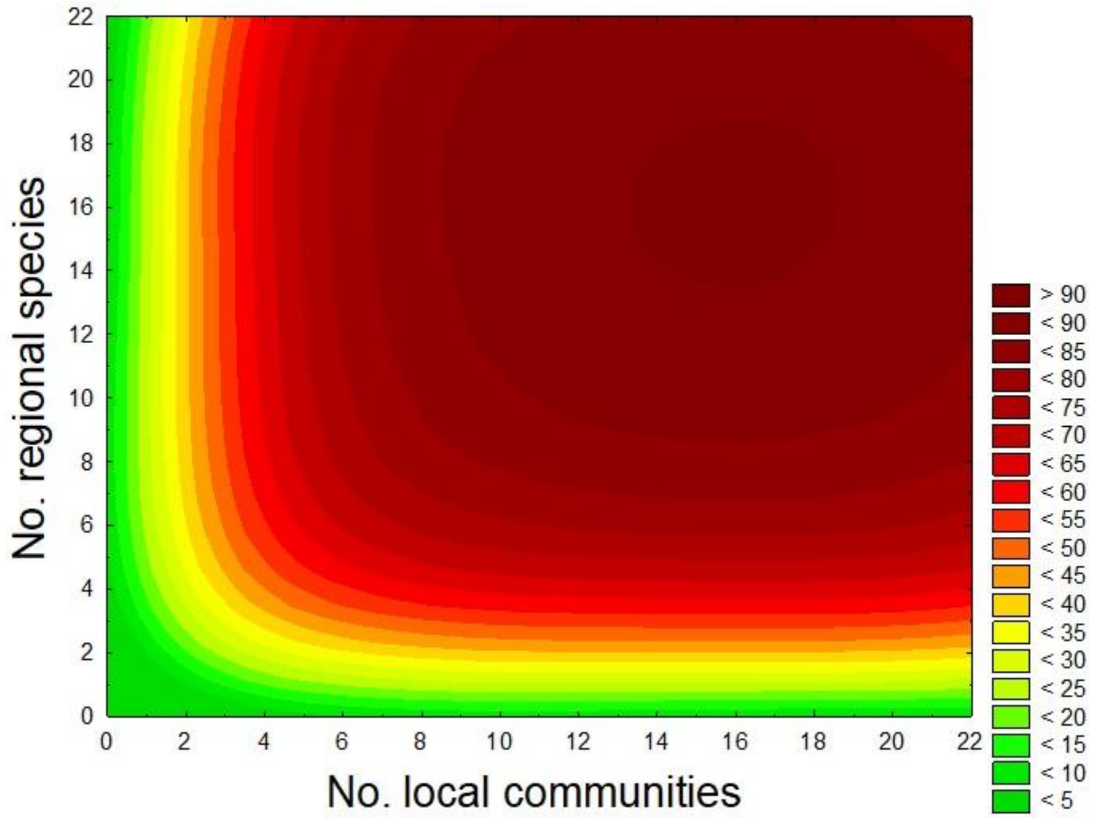

Figure S1. Percent of all population pairings that are cross-community (i.e., different species living in different local communities) as a function of number of local communities and regional richness.

Literature cited

Doak, D. F., D. Bigger, E. K. Harding, M. A. Marvier, R. E. O'Malley, and D. Thomson. 1998. The statistical inevitability of stability-diversity relationships in community ecology. The American Naturalist 151:264–276.

Tilman, D., C. L. Lehman, and C. E. Bristow. 1998. Diversity-stability relationships: Statistical inevitability or ecological consequence? The American Naturalist 151:277–282.
